# Abnormalities in intron retention characterize patients with systemic lupus erythematosus

**DOI:** 10.1038/s41598-023-31890-4

**Published:** 2023-03-29

**Authors:** Xiaoqian Sun, Zhichao Liu, Zongzhu Li, Zhouhao Zeng, Weiqun Peng, Jun Zhu, Joel Zhao, Chenghao Zhu, Chen Zeng, Nathaniel Stearrett, Keith A. Crandall, Prathyusha Bachali, Amrie C. Grammer, Peter E. Lipsky

**Affiliations:** 1 Computer Science Department, George Washington University, Washington, DC 20052 USA; 2 Physics Department, George Washington University, Washington, DC 20052 USA; 3 Mokobio Biotechnology R&D Center, 1445 Research Blvd, Suite 150, Rockville, MD 20850 USA; 4 Walt Whitman High School, Bethesda, MD 20817 USA; 5 Mclean High School, McLean, VA 22101 USA; 6 Computational Biology Institute, Milken Institute School of Public Health, George Washington University, Washington, DC 20052 USA; 7 RILITE Research Institute and AMPEL BioSolutions, 250 W Main St, Ste 300, Charlottesville, VA 22902 USA

**Keywords:** Computational biology and bioinformatics, Genetics, Immunology, Biomarkers, Diseases, Molecular medicine, Rheumatology

## Abstract

Regulation of intron retention (IR), a form of alternative splicing, is a newly recognized checkpoint in gene expression. Since there are numerous abnormalities in gene expression in the prototypic autoimmune disease systemic lupus erythematosus (SLE), we sought to determine whether IR was intact in patients with this disease. We, therefore, studied global gene expression and IR patterns of lymphocytes in SLE patients. We analyzed RNA-seq data from peripheral blood T cell samples from 14 patients suffering from systemic lupus erythematosus (SLE) and 4 healthy controls and a second, independent data set of RNA-seq data from B cells from16 SLE patients and 4 healthy controls. We identified intron retention levels from 26,372 well annotated genes as well as differential gene expression and tested for differences between cases and controls using unbiased hierarchical clustering and principal component analysis. We followed with gene-disease enrichment analysis and gene-ontology enrichment analysis. Finally, we then tested for significant differences in intron retention between cases and controls both globally and with respect to specific genes. Overall decreased IR was found in T cells from one cohort and B cells from another cohort of patients with SLE and was associated with increased expression of numerous genes, including those encoding spliceosome components. Different introns within the same gene displayed both up- and down-regulated retention profiles indicating a complex regulatory mechanism. These results indicate that decreased IR in immune cells is characteristic of patients with active SLE and may contribute to the abnormal expression of specific genes in this autoimmune disease.

## Introduction

Systemic lupus erythematosus (SLE) is a complex, multisystem autoimmune disease affecting many different organs^1,2^. It is characterized by excessive production of antibodies against self-proteins and nuclear material and dysregulation of T and B cell function^3–5^. For each individual patient, the disease phenotype may vary from relatively mild manifestations to life- threatening organ damage^6^.

The occurrence of SLE is heavily influenced by genetics with a heritability of 66%^7,8^. Whereas genetic factors confer a predisposition to the development of SLE^1,9^, and in few cases even single gene deficiencies may lead to SLE, genetic determinants of SLE severity are elusive because of the high genetic heterogeneity associated with the disease^10,11^. It is currently accepted that SLE is a result of the combined and cumulative effect of variants in a large number of genes, as well as environmental influences^2^. Although numerous genetic variants have been identified by genome-wide association studies (GWAS) that contribute to the risk of SLE^8^, the effect of the majority of the risk alleles is still unknown^12^. Strikingly, among the numerous single-nucleotide polymorphisms (SNPs) associated with SLE, most reside in non-coding DNA regions^1,13^ and are thought to affect gene regulation^12^. This indicates that the non-coding information, either by controlling the gene expression level or acting in some other regulatory manner, may influence development of this disease.

Considerable attention has been devoted to the analysis of the contribution of epigenetic regulation in SLE^13,14^. Although there are numerous mechanisms that influence gene expression, the role of intron retention (IR) and its regulation have been recently identified as the basis of alternative splicing as well as the final steps in gene expression. IR has been found to progressively accumulate in diverse human and mouse tissues during development^14–17^. The functional implications of IR in gene regulation were not fully appreciated until recently^18–20^. The influence of IR on gene regulation has been identified in higher eukaryote processes, such as neurogenesis^21^, granulocyte differentiation^18^ and terminal erythropoiesis^22,23^. Recent studies have also shown that gene expression regulated by global IR may play a role during CD4 T cell activation^24^. However, previous studies have not explored the role of IR in SLE.

Spliceosomes serve to remove noncoding sequences from precursor mRNA and ligate coding sequences into functional mRNA molecules^25,26^. Each human cell contains approximately 100,000 spliceosomes, each composed of ~ 300 different proteins and RNAs^27^. Many human diseases are caused by errors in either splicing of a single gene, which account for around 35% of human genetic disorders, or regulation of the entire spliceosome as a result of mutations of spliceosomal proteins themselves^27,28^. SLE and many other autoimmune diseases, such as scleroderma, multiple sclerosis and myasthenia gravis, are reported to have abnormalities in splicing resulting in increased or aberrant alternative splicing^29^. Specifically, it has been recognized that splicing factor perturbation has a broad impact in SLE^30^, especially in T cells^31,32^.

Based on these considerations, we studied the global gene expression and IR patterns of purified lymphocytes from SLE patients. Decreased IR was found in SLE that correlated with disease activity in the studied datasets. The consistency of association between whole gene expression, spliceosomal protein gene expression and IR pattern indicates an inherent regulatory correlation among them. The results are consistent with a diffuse abnormality in IR in active SLE that may contribute to the dysregulated gene expression pattern characteristic of this autoimmune disease.

## Methods

### RNA sequencing and IR analysis

We analyzed two distinct datasets for the occurrence of intron retention to see if the phenomenon occurred in both T cells and B cells. The first RNA-seq data were from peripheral blood T cell transcriptomes of 14 Lupus patients (12 females and 2 males) and 4 healthy controls downloaded from the NCBI Sequence Read Archive with Bioproject Accession ID PRJNA293549^2^. Seven SLE samples were from patients whose SLE was not currently active (SLE Disease Activity Index, SLEDAI < 7), and two were from patients whose SLE are highly active (L149 with SLEDAI = 20, L074 with SLEDAI = 26). TopHat2^33^ was used to process FastQC files (https://www.bioinformatics.babraham.ac.uk/projects/fastqc/) from Illumina sequencing which provided approximately 75e6 paired 90 bp reads for each sample. More detailed information, such as sex, age and SLEDAI, for each SLE sample can be found in Table 1 of Bradley et al.^2^, and demographic information for each control can be found in Table S1 showing controls were age and sex matched to SLE cases. The second RNA-seq data were from B cell subsets (CD11c hi IgD + B cells, CD11c hi IgD-B cells, Memory B cells and Naïve B cells) of 4 healthy controls and 16 Lupus patients (distinct from the T cell patient population) downloaded from the NCBI Sequence Read Archive with Bioproject Accession ID GSE110999^14^, with 50 million average read count per pair. Sequencing reads were aligned to human reference genome hg19 using Hisat2 (v2.0.2)^34^ and STAR^35^ 2.5.2a for sorted SLE B cell subsets and sorted healthy subjects, respectively. This is one potential cause of inconsistency in control samples, and so, 4 control samples with relative larger number of identified genes were selected for further analysis. Demographics and clinical characteristics of healthy donors and lupus patients was summarized in Supplementary Table 1 of Wang et al.^14^.

Paired-end mapping of the RNA-seq data using TopHat2^33^ with default parameters with respect to human genome hg19 from Illumina was carried out. Expression scores of uniquely mapped reads in units of reads per kb per million (RPKM) were obtained by CUFFLINKS^36^ for the 26,372 best annotated genes, including expressed pseudogenes and noncoding RNAs. To reliably evaluate the levels of IR, we adopted the Intron Retention Index (IRI) metric using IRTools (https://pypi.org/project/IRTools/) that has been successfully applied in the analysis of human CD4 T cell activation^23^. IRTools provides two complementary metrics to enhance consistency of IR analysis, i.e., intron retention index (IRI) and intron retention coefficient (IRC). The former uses sequence reads from exonic and intronic regions, whereas the latter only junction reads. In this study, we utilized IRI as the primal metric. Since IRTools only supports human genome hg19, all data processing was done with respect to hg19. The IRI of a gene was defined as the ratio of its read density of shared intronic regions and that of shared exonic regions. Specific criteria and cutoffs follow those previously described^23^. Only genes with high expression level, i.e., RPKM > 1, were used for the IRI evaluation to reduce the statistical error by excluding genes of very low expression levels. This resulted in ~ 8000 genes for each sample with an overall overlap of 7645 genes for T-cell samples, and ~ 8000 genes for each sample with an overlap of 3621 genes for B-cell samples, and 3475 common genes between T-cell and B-cell samples.

### Unbiased hierarchical clustering analysis

Unbiased hierarchical clustering of samples, including patients and controls, was performed on three different metrics: expression level of all overlapping genes, expression level of only spliceosomal genes, and IRI level of all overlapping genes. To standardize the broad range of values across the samples in either gene expression or IRI levels, we used the Z-score for clustering. Z-score of a level value $V_{i}^{s}$ for *i*-th gene in sample *s* was calculated as follows: $Z_{i}^{s} = \frac{{V_{i}^{s} - \mu_{i} }}{{s_{i} }}$, where $\mu_{i}$ and $s_{i}$ stand for the sample average and standard deviation of the level value $V_{i}^{s}$, i.e., expression or IR level, of *i*-th gene over $N_{s}$ samples as $\mu_{i} = \frac{1}{{N_{s} }}\mathop \sum \limits_{s}^{ } V_{i}^{s}$ and $s_{i}^{2} = \frac{1}{{N_{s} - 1}}\mathop \sum \limits_{s}^{ } \left( {V_{i}^{s} - \mu_{i} } \right)^{2}$. Hierarchical clustering was implemented with Python package Seaborn^37^ with both the ‘mean’ linkage method and ‘Euclidean’ distance metric applied to the gene expression or IRI dataset.

### Principal component analysis (PCA) of gene expression level and intron retention index

Principal modes of sample-sample variation were obtained by decomposing the covariance matrix *C*, an $N_{g} \times N_{g}$ matrix with its element $C^{{\left( {ij} \right)}}$ being the covariance of gene expression $g_{i}^{s}$ and $g_{j}^{s}$ for *i*-th and *j*-th genes, respectively, out of a total of $N_{g}$ genes in samples. The deviation from the sample mean is denoted as $\Delta g_{i}^{s} = g_{i}^{s} - \left\langle {g_{i}^{s} } \right\rangle$ where the bracket stands for sample mean. The covariance was calculated by the equation, $C^{{\left( {ij} \right)}} = \left\langle {\Delta g_{i}^{s} \Delta g_{j}^{s} } \right\rangle$ where $1 \le i,j \le N_{g}$ and $1 \le s \le N_{s}$. Here $N_{s}$ is the number of samples. For example, in the T-cell dataset, $N_{s} = 18$ for 14 SLE patients plus 4 controls. In the case of IRI analysis, the above $g_{i}^{s}$ was replaced by the IRI value for the i-th gene in sample *s*. Principal components were then obtained by diagonalizing the covariance matrix: $C = \mathop \sum \limits_{k = 1}^{{N_{g} }} \sigma_{k} P_{k} P_{k}^{T}$, where $\sigma_{k}$ and $P_{k}$ are the respective *k*-th eigenvalue and eigenvector (also called the Principal Component) of *C*. Symbol *T* above denotes transpose. The fractional contribution of $P_{k}$ to sample variation in the dataset is given by $f_{k} = \sigma_{k} /\mathop \sum \limits_{k}^{ } \sigma_{k}$ are ranked according to their relative contribution to the total variance and only the top 2 or 3 are retained to approximate the whole $N_{g}$-dimension space.

### Gene-disease enrichment analysis and gene-ontology enrichment analysis

The first and dominant principal component (PC1) from PCA analysis of transcriptomic information, such as intron retention, clearly distinguished SLE patients from controls. To gain further insight on the possible phenotypic implications of PC1, possible enrichment of lupus related disease and RNA processing related ontologies were examined for two subsets of genes relative to the whole gene set. Since PC1 is a weighted linear sum of all genes, two subsets of genes, i.e., those with the highest positive coefficients and those with highest negative coefficients in PC1, respectively, were assessed. This is based on the assumption that samples with the highest positive coefficients in PC1 contain genes whose IRI were suppressed in SLE patients relative to the controls, whereas those with the negative coefficients were enhanced. In both T and B cell analyses, 50 genes with the most negative coefficients and 100 genes with the most positive coefficients were selected. For cross-reference to genes enriched in lupus related diseases, known disease types associated with each gene in the whole set were obtained from the DisGeNET database^38^. For assessment of genes related to RNA processing ontologies, gene sets were acquired from Enrichr^39–41^ by providing subset gene lists and whole set gene lists. Enrichment of a specific disease/ontology of interest was evaluated by calculating the probability of at least $k$ genes (genes that are associated with the relevant disease/ontology) in the subset (containing $n$ genes) from the whole genome of population size $N$ that contains $K$ genes with the same feature by Hypergeometric Testing using SciPy^42^ in Python.

### Differential intron retention significance level analysis

To analyze the intron retention profile globally and specifically, the splicing ratio of each intron was calculated. The splicing ratio of an intron is defined as the ratio between the RPKM of the intron and the averaged RPKM of two flanking exons^43^. The splicing ratio of each intron from SLE patients and controls was compared. The Mann–Whitney U test was applied to quantify the significance (p-value) of the group difference between SLE patients and controls (i.e., 14 SLE vs. 4 control samples for T cells and 16 SLE vs. 4 control samples for B cells). All introns were ranked in volcano plots according to their statistical p-value and their relative difference of the splicing ratio. The retention difference of a specific intron between SLE patients and controls was also analyzed and presented in the box plots.

## Results

### Abnormal intron retention in T and B cells from SLE patients

**Figure 1:**
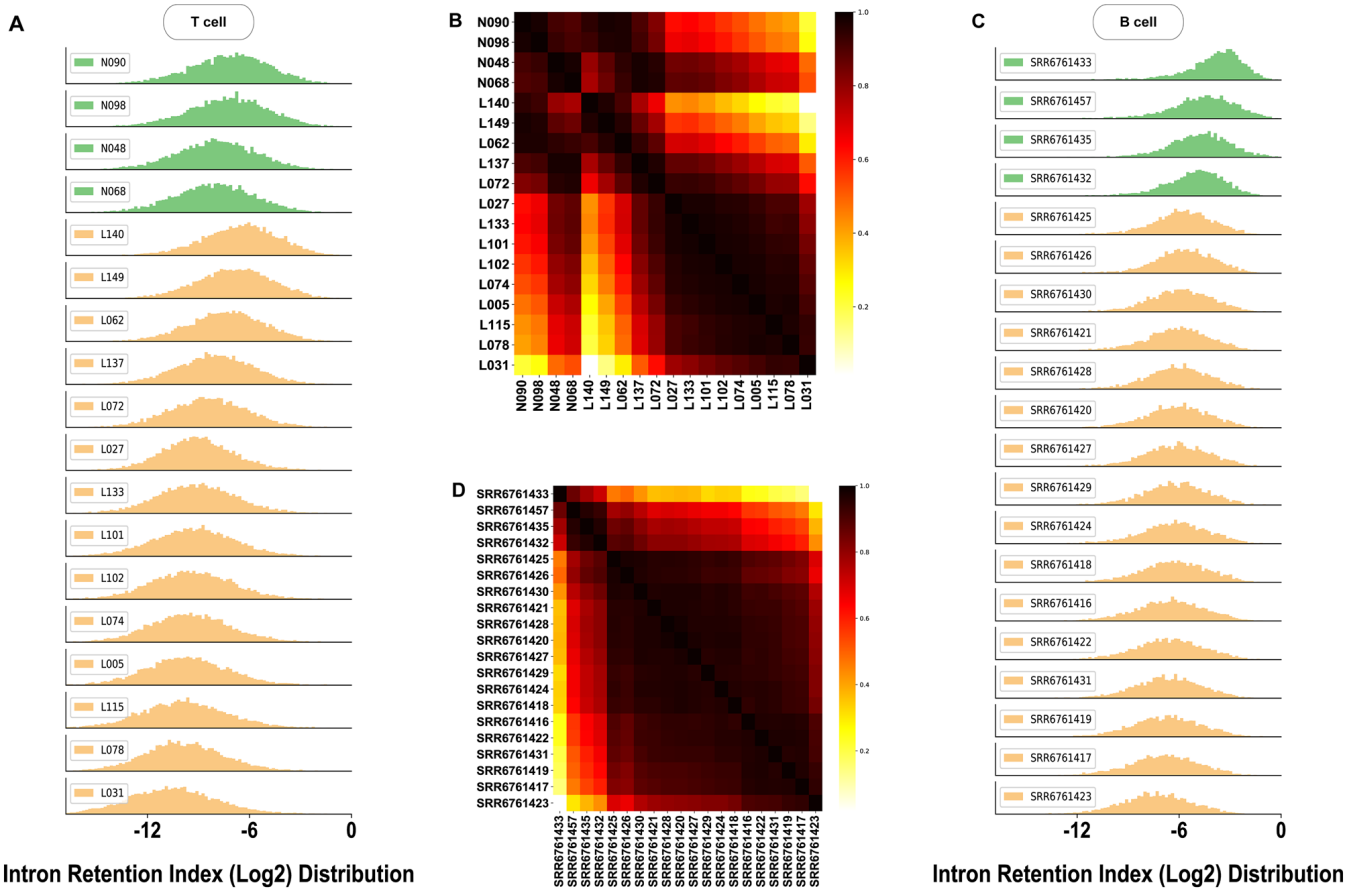
Intron retention index (IRI) in T and B cells. (**A**) The density distribution of IRI from all 18 T-cell samples including 4 control samples (green) and 14 SLE patients (gold). (**B**) Heatmap of pairwise Pearson’s correlation coefficient matrix of IRI distributions shown in (**A**). There appeared to be two major diagonal blocks separated around L137 with a majority of SLE patients forming the lower block. (**C**) The density distribution of IRI of all 20 B-cell samples, including 4 control samples (green) and 16 SLE patients (gold). Note that the B cell samples were obtained from a cohort different from that for T cells in (**A**) above. Namely, T-cell and B-cell samples were not from the same cohort. (**D**) Heatmap of pairwise Pearson’s correlation coefficient matrix of IRI distributions shown in (**C**). The lower diagonal block formed by SLE patient samples was clearly separated from the upper block formed by control samples.

To probe the difference in intron retention between SLE patient samples and control samples, we calculated each individual gene’s intron retention index (IRI) from RNA-Seq data from CD4 + T cells collected from 14 SLE patients and 4 control samples^1^. The histogram shown in Fig. 1A was obtained from 7645 genes with detectable IRIs in all 18 samples. According to the pairwise Pearson’s correlation coefficients between any two IRI distributions (Fig. 1B), the 18 samples appeared to be separated into two major diagonal blocks. The upper block contained all 4 control samples and 4 SLE patients (L140, L149, L062, and L137) and retained a higher level of IRI with a mean value of Log2(IRI) =  − 6.0, whereas the majority of all other SLE patient samples in the lower block had very similar distribution to each other with considerably reduced IRI, a mean value of Log2(IRI) = − 8.0 (see Fig. S1 and Table S2 for detailed summary of statistical attributes of these histograms). Notably, IRI distributions in T cells from SLE patients appeared to separate into two diametrically opposite subgroups: a majority with significantly decreased IR relative to the controls and four others (i.e., L140, L149, L062, and L137) with IR comparable to normal or even enhanced. SLEDAI scores were quite high, 20 and 16, on L149 and L137, respectively. L149 and L140 were the only two male patients. Mean distributions of control and SLE IRI distributions can be found in Fig. S3A with p-value equals to 0.079 using Mann–Whitney U test.

Since dysregulation of B cell function is believed to be associated with SLE pathogenesis^44^, sample stratification using IRIs was also carried out in sorted B cells from an independent cohort of SLE patients. Figure 1C shows the histograms of IRI distribution for each sample obtained from 3621 genes with IRIs detectable in all 20 samples (4 control samples and 16 SLE patient samples). A similar global shift toward reduced IRI levels was also observed in SLE B cells (Fig. S2 and Table S3), supported by distinguished mean distributions (Fig. S3B) (*p* value = 0.0004 using Mann–Whitney U test) of control and SLE IRI distributions. Based on the pairwise Pearson’s correlation coefficients between two IRI distributions in Fig. 1D, the 20 samples were clearly divided into two blocks, 4 control samples in one block and 16 SLE samples in the other block. Interestingly, in contrast to the pattern of two distinct SLE subgroups based on IRIs of T cells, B cells in all SLE patients showed a remarkably converged pattern centered around a mean value of Log2(IRI) =  − 5.2, which was decreased from a value of Log2(IRI) =  − 3.9 for the control samples.

### Weak and negative correlation between fold changes in gene expression and intron retention index in T and B cells

**Figure 2:**
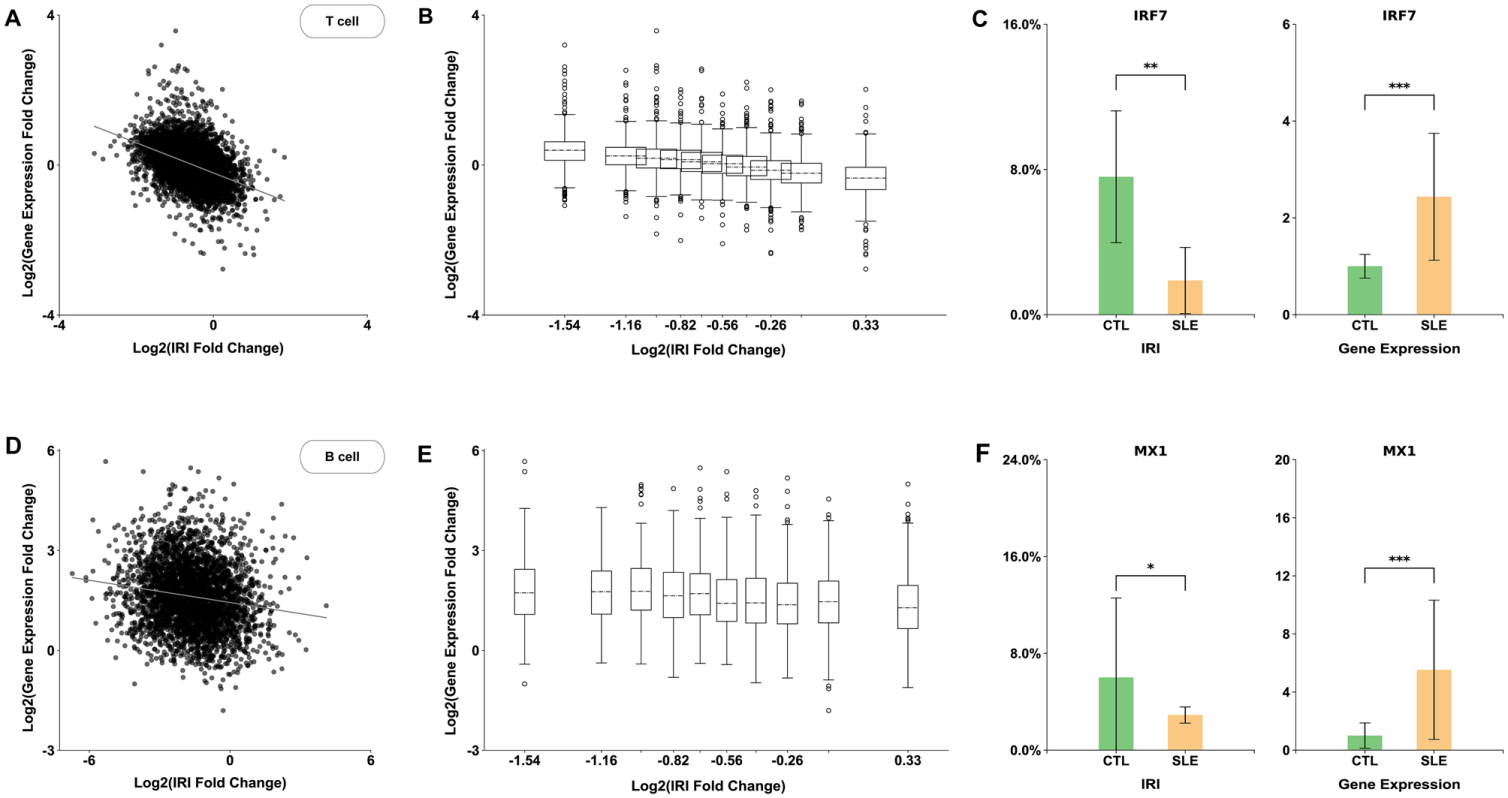
Gene expression level is associated with IR in T and B cells. (**A**) The scatter plot of fold change in gene expression vs. the fold change of IRI with a Pearson’s correlation coefficient of − 0.34 (*p* < 5E − 200) for T cells. (**B**) The box plot of (**A**) where genes were evenly divided into 10 bins of equal numbers according to the sorted IRI fold changes. The fold changes in gene expression for each bin were shown as a box plot along the y-axis and the average fold change in IRI for each bin was labeled along the x-axis. (**C**) Fold change in IRI and gene expression for a specific gene IRF7 as an illustrative example. A decreased IRI level (left panel) occurred with an increased gene expression level (right panel) for IRF7 in SLE patients relative to the controls (CTL). (**D**) Same as (**A**) but for B cells with a Pearson’s correlation coefficient of − 0.062 (*p* = 0.0019). (**E**) Same as (**B**) but for B cells. (**F**) Same as (**C**) but for B cells with a specific gene MX1 as an example showing the opposite fold changes in IRI and gene expression.

Given that the observed large-scale IR modifications above and the previously reported gene expression anomaly^45,46^ were both associated with SLE pathogenesis, it is natural to examine the relation between IR and gene expression. As a quantitative measure, we computed the fold change defined as the ratio of the mean value of either IRI or gene expression level between SLE patients and the controls. Figure 2A and B clearly showed a weak but significant correlation between IRIs and gene expression levels in T cells as confirmed by the negative Pearson’s correlation coefficient (r =  − 0.34, *p* < 5E-200). As further illustrated by the case of a specific gene, i.e., interferon regulated factor 7 (IRF7), up-regulated expression of IRF7 in SLE patients (relative to the controls) was accompanied by a down-regulated IRI of the IRF7 transcript as shown in Fig. 2C. This pattern between IR and gene expression was consistent with the earlier results reported during in vitro T-cell activation^23^. Using ChIP-Seq of RNA Pol II or histone mark H3K36me3 as a proxy for transcription activity, IR was found to be associated with transcript instability^23^. This result was further validated experimentally on a genome-wide scale in CD4^+^ T cells^47^. Similar results were also obtained for B cells (Figs. 2D, E, and F) with an even weaker negative correlation coefficient (r =  − 0.062, *p* = 0.0019). Whereas the statistically significant correlations strongly supported the conclusion that gene expression levels were regulated by intron retention, the weak correlation coefficients seen in the scatter plots (Fig. 2A and D) were suggestive that the average metric of IRI, which sums over all intron reads and thus treats all introns within a gene equally, was insufficient to capture a more complex relationship between gene expression and intron retention.

### Enrichment of RNA processing function among genes with significant IRI modifications

**Figure 3:**
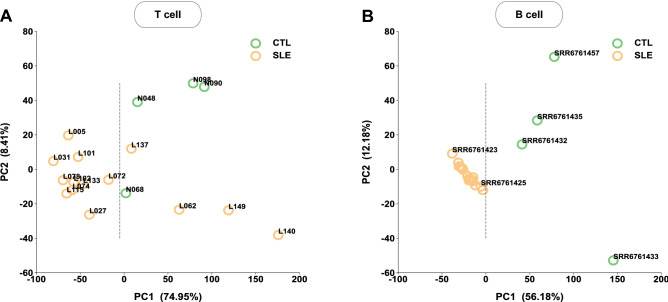
Principal component analysis (PCA) on IRI for T-cell and B-cell samples. (**A**) The 18 T-cell samples were projected onto the top two components (PC1 and PC2) with PC1 covering 74.49% of sample variation and PC2 8.41%. Samples projected onto positive or negative directions of PC1 were delineated by a vertical dashed line. (**B**) Similar results for the 20 B-cell samples where PC1 explained 56.18% of sample variation and PC2 12.18%. The projection onto the positive and the negative directions along PC1 separated, respectively, the control samples (CTL, green) from either the majority SLE samples (SLE, gold) for T cells or all the SLE samples (SLE, gold) for B cells.

The frequency histogram (Fig. 1A and C) depicting the global IR reduction in SLE patients counted the number of genes with IRI values within certain ranges without identifying specific genes among either T-cell or B-cell samples. To examine further the role that IR played, we focused on specific genes or their combinations that were common yet significant in characterizing IRI variations among samples. To this end, we performed principal component analysis (PCA) on standardized IRIs over 18 T-cell samples. The top two principal components (PC1 and PC2) together captured a large percentage (83.36%) of the total IRI sample variation, with 74.49% from PC1 and 8.41% from PC2. Similar results were also obtained for 20 B-cell samples. As shown in Fig. 3, PC1 alone largely separated the control samples from SLE samples for both T and B cells. This is important since it implies that insight on the interplay of IR and SLE may be gained by focusing on PC1 only. We thus analyzed the possible enrichment in certain biological functions among the most distant genes in PC1 only, i.e., those genes with the largest absolute value of coefficients in PC1. For this purpose, 50 genes with the most negative coefficients and 100 genes with the most positive coefficients in PC1 for T or B cells were selected (Table S4). Since majority genes (~ 90%) fall ln PC1 positive side, less genes (50) were picked from negative side and more genes (100) were picked from PC1 positive side. The potential enrichment of either biological functions or lupus related diseases in this selected set relative to the entire gene set were examined via a hypergeometric distribution score. The results showed that the biological functions of RNA processing were significantly enriched (*p* < 0.05) among the “book end” genes for both T and B cells (Table S6), whereas the enrichment in Lupus related diseases was only observed for B cells (Table S5). It was also notable that these book end genes had almost no overlap between T and B cells, indicating possibly distinctive or sequential roles of IR in T and B cells in SLE pathogenesis.

### Similar sample clustering by gene expression of the whole genome and the splicing factors

**Figure 4:**
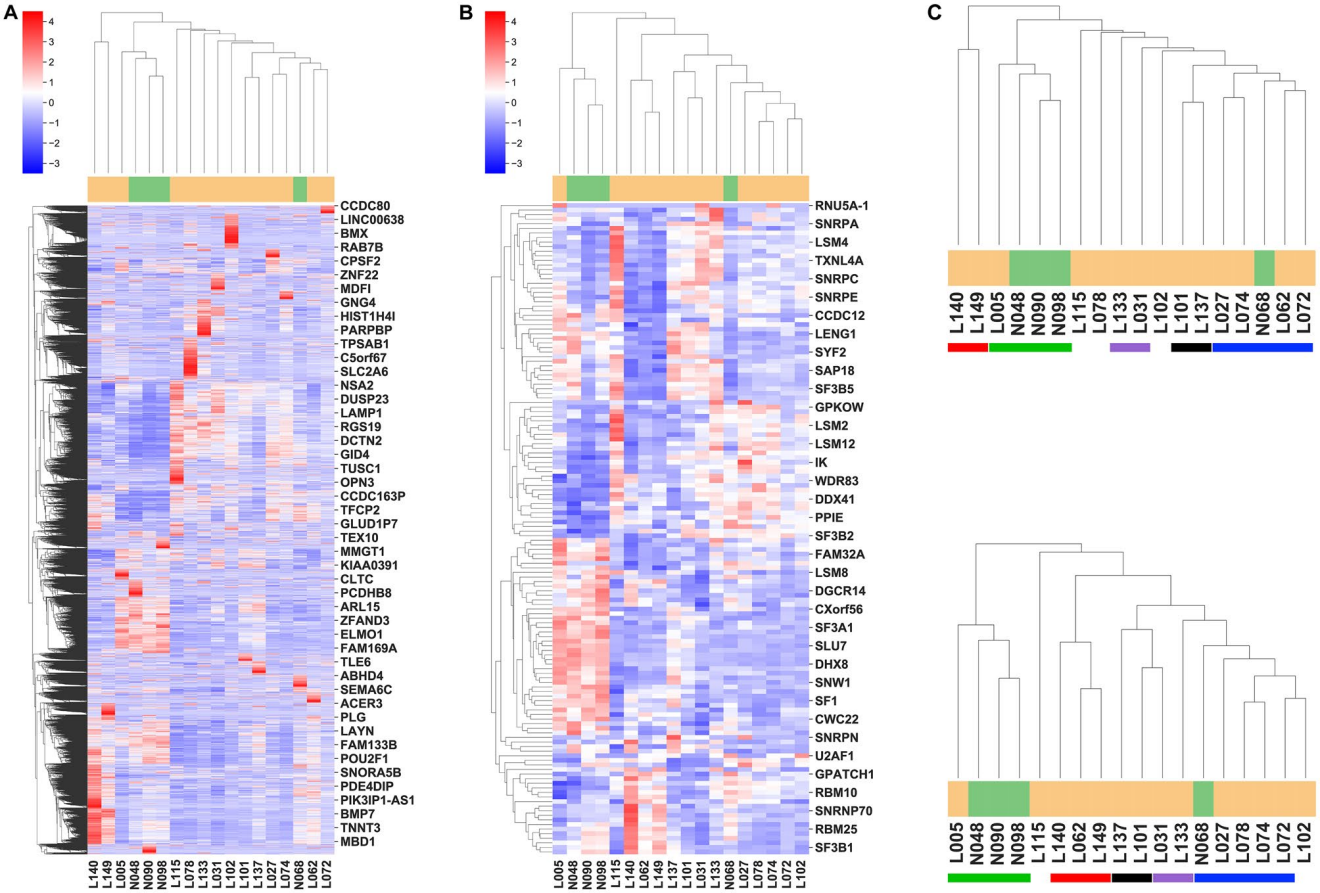
Gene expression profiles at both the genome level and the splicing factors level for T-cell samples. (**A**) Gene expression based hierarchical clustering of all genes in the whole transcriptome of all 18 T-cell samples including 4 normal controls and 14 SLE patients. Sample clustering is shown at the top with the sample label at the bottom. Gene clustering is shown to the left and ~ 50 marker genes distributed uniformly on the right side as location reference to gene clustering in Table S7. (**B**) Same clustering as in (**A**) but using 142 splicing factor genes (from HUGO Gene Nomenclature Committee Database) instead of the whole genome. About 40 marker genes on the right were distributed uniformly as location reference to splicing factor clustering in Table S8. (**C**) Similar clustering patterns of sample stratifications between the whole genome gene expression (top) and the splicing factor gene expression (bottom) were indicated by the color bars with the same color for the same pattern.

Given that enriched RNA processing was observed above among the book end genes involved in global IR modification and IR was correlated with gene expression (Fig. 2), we reasoned that the gene expression profile at the whole genome level would be closely linked to the gene expression profiling of splicing factors. To test this link, we compared the sample clustering of T cells according to gene expression at either the whole genome level (Fig. 4A) or that of splicing factors (Fig. 4B). As shown in Fig. 4C, several exact or similar patterns among the 18 samples were found in common between these two gene expression profiles, i.e., (L140, L149), (L005, N048, N090, N098), (L133, L031), (L101, L137), and (L027, L074, N068, L072). These overlaps suggested that the small number of splicing factors were the effective mediators linking IR and gene expressions and they could be used to achieve effective dimensionality reduction of the whole transcriptome expression for SLE patient stratification. Hieratical clustering heatmaps and similarities in sample clustering for B-cell sample using both genome level gene expression and the splicing factors level gene expression are available in Fig. S4, which is consistent with the finding in T-cell samples.

### Complex regulation of intron retention at individual intron level within a given gene

**Figure 5:**
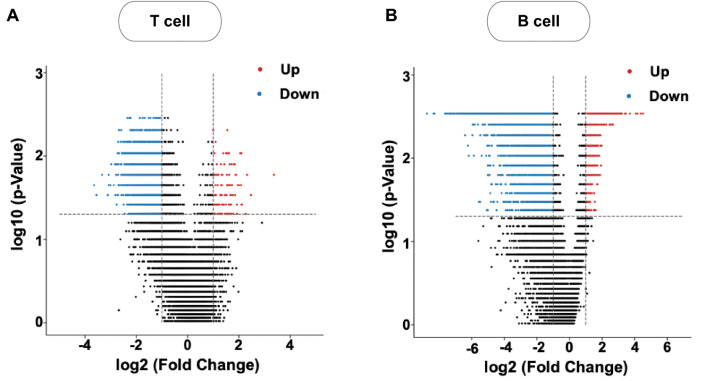
Volcano plots summarizing the comparison of the splicing ratio of individual intron between SLE patients and control samples in T cells (**A**) and B cells (**B**). Fold change of splicing ratio between SLE and control samples was computed and displayed along the x-axis in Log2 scale. Permutation-based *p* values on the significance of fold change were computed via Mann–Whitney U test and displayed along the y-axis in Log10 scale using 14 SLE versus 4 control samples for T cell and 16 SLE versus. 4 control samples for B cells. Each dot is for one intron. Those introns with |Fo\ldChange|≥ 2.0 (x-axis) and *p* value ≤ 0.05 (y-axis), i.e., those with significantly altered splicing ratio, were color coded with red for up-regulated (SLE vs. control samples) and blue for down-regulated. Specifically, for T cells in (**A**), there were 5356 blue dots (from 2617 unique genes) and 120 red dots (from 106 unique genes) out of 30,319 dots in total; and for B cells in (**B**), 5754 blue dots (from 2839 unique genes) and 533 introns (414 unique genes) out of 11,114 dots.

Since it is known that splicing factors target specific introns within a gene^43^, it is necessary to focus on the intron retention at each individual intron. To do this, we calculated the splicing ratio of individual introns, i.e., the ratio between reads of a given intron and the average reads of its two flanking exons, and further identified those introns with significant fold change in splicing ratio between SLE and control samples. The results for T and B cells are shown in Fig. 5. The intron splicing ratio profiles at the individual intron level showed a similar pattern for both T and B cells with a dominant downregulation in splicing ratio for SLE patients as blue dots greatly exceeded the red dots in Fig. 5. This was rather consistent with the earlier observation of a downshift in the global IRI histograms in Fig. 1.

Notably, the down-regulated and up-regulated introns shared 18 and 226 common genes for T and B cells, respectively (see Tables S9 and S10 for the list of such genes and introns). Therefore, in both T and B cells, introns within individual genes could exhibit both increased and decreased retention, implying that they might be differentially regulated by unique splicing factors. Among the genes that contain both up- and down-regulated introns, the first intron has a higher probability to be up-regulated in T cells (Table S9, Fig. S5A) and down-regulated for B cells (Table S10, Fig. S5B), which is consistent with previous studies on the functional role that the ordinal position of introns may play in intron retention^48^.

## Discussion

The present study did not address the potential interplay between T and B cells in SLE pathogenesis since T cells and B cells were collected from different cohorts. It instead focused on the interplay between the spectrum of intron retention, global gene expression and up-regulation of splicing factors in the context of SLE pathogenesis. We found that SLE samples were characterized by both enhanced expression of splicing factors and decreased global intron retention, and that these perturbations were associated with an increase in global gene expression. These results suggest the hypothesis that a fundamental abnormality in IR mediated by increased expression of splicing factors could contribute to the abnormal gene expression characteristic of SLE.

IR appears to be the final step in gene expression and plays a role in many stages of cell differentiation^49^. IR regulates gene expression by a number of mechanisms, including the premature termination of translation and, because introns often contain premature termination codons, the induction of nonsense mediated mRNA decay. A role for abnormal IR has been suggested in a number of diseases, including neurodegenerative conditions, cancer and Duchenne muscular dystrophy, but a role in inflammatory/autoimmune diseases has not previously been suggested. Here, we clearly showed evidence of decreased IR in both T and B lymphocytes of patients with the autoimmune/inflammatory disease, SLE. Since the abnormality in IR was global, it is possible that decreased IR facilitates gene expression of numerous genes in immune cells in this condition, thereby removing an important element of the control of gene expression in SLE and facilitating many of the immunoregulatory abnormalities characteristic of this condition.

Among the abnormalities noted in SLE was a decrease in IR in specific introns in IRF7, a molecule involved in regulating the interferon response. Abnormalities in IRF7 expression and the interferon gene signature are characteristic of SLE^49^ and the decrease in IR within the IRF7 gene may contribute to this. It is noteworthy that previous studies have examined the fine specificity of the regulation of IR within IRF7 and noted specific factors that regulate the IR in intron 4 of this mRNA^43^. It is likely that other mechanisms are regulating IRF7 IR in SLE as BUD13 was not upregulated and intron 4 was not differentially subjected to IR.

It was previously reported that the global intron retention profile for T lymphocytes was reduced following in vitro activation^23^. Here, we show that IR was decreased in both T and B cells from SLE patients by a comparable degree. Since evidence suggests that both T cells and B cells are activated in SLE in vivo^1,50^, the current results are consistent with the conclusion that the decreased IR in both lymphocyte populations reflects the activation status of the cells.

Adding to the numerous abnormalities in T and B lymphocytes in SLE^50^, the significant reduction in IR represents another regulatory abnormality as an important mechanism of gene expression control. It is notable that despite the significant decrease in IR in both T and B cells in SLE, there was almost no overlap in individual introns in T and B cells that were significantly and differentially retained in SLE patients, indicating distinct regulatory abnormalities in the two lymphocyte populations. Further work will be necessary to investigate the implications of these abnormalities on cellular function in detail.

It is clear that regulation of IR is complex, with many splice factors being involved^51^. This likely contributed to the rather weak correlation observed between global gene expression and IRI. This is partly because IRI averages all introns within a gene and does not consider the retention profile of each individual intron and the structural features of each gene that influence IR, including, intron length and ordinal position. Notably, regulation of IR within the same genes was found to be complex, with some introns regulated comparably, but many introns regulated in opposite manners, suggesting a complex regulation network whose decoding will require additional analysis.

## Conclusion

IR is an important regulatory mechanism of alternative splicing that differentiates SLE from healthy individuals. The data suggest a role for dysregulated IR in abnormalities in global and specific gene expression in SLE that could reflect differences in cellular activation status. Moreover, abnormal up-regulation of IR and expression of splice factor genes may play an important role in the global dysregulation of IR in SLE. Further delineation of IR in SLE may provide additional insights into the abnormal control of gene regulation in this condition and also provide new targets of therapeutic interventions.

## Supplementary Information

Supplementary Information 1.

Supplementary Figure S1.

Supplementary Figure S2.

Supplementary Figure S3.

Supplementary Figure S4.

Supplementary Figure S5.

Supplementary Table S1.

Supplementary Table S2.

Supplementary Table S3.

Supplementary Table S4.

Supplementary Table S5.

Supplementary Table S6.

Supplementary Table S7.

Supplementary Table S8.

Supplementary Table S9.

Supplementary Table S10.

## Data Availability

Bioproject Accession ID PRJNA293549—peripheral blood T cell transcriptomes from 14 Lupus patients (12 females and 2 males) and 4 healthy controls. Bioproject Accession ID GSE110999—RNA-seq data from B cell subsets from 16 Lupus patients and 4 healthy controls. Publicly available data from NCBI – accessions provided in Methods.

## Abbreviations

SLE: Systemic lupus erythematosus
IR: Intron retention
GWAS: Genome-wide association study
SNP: Single nucleotide polymorphism
SLEDAI: SLE disease activity index
RPKM: Reads per kilobase per million
IRI: Intron retention index
PCA: Principal component analysis
