# Supplementary material for: Abnormalities in intron retention characterize patients with systemic lupus erythematosus
Source: Sci Rep. 2023 Mar 29;13:5141. doi: 10.1038/s41598-023-31890-4 (PMC10060252; doi:10.1038/s41598-023-31890-4)
Supplement: Supplementary file 1 — Supplementary Information 1. [file 41598_2023_31890_MOESM1_ESM.docx]

**List of Supplementary Website and Documents**

**Abnormalities in Intron Retention Characterize Patients with Systemic Lupus Erythematosus**

Xiaoqian Sun, Zhichao Liu, Zongzhu Li, Zhouhao Zeng, Weiqun Peng, Jun Zhu, Jole Zhao, Jimmy Zhao, Chen Zeng, Nathaniel Stearrett, Keith A. Crandall, Prathyusha Bachali, Amrie C. Grammer and Peter E. Lipsky

**Inventory:**

**Visualization of intron reads among T and B cells:** http://lupusigv.landscapesun.com

**Figure S1: IRI distribution comparison of T-cell samples.**

**Figure S2: IRI distribution comparison of B-cell samples.**

**Figure S3: Mean distribution of IRI distributions of T-cell and B-cell samples.**

**Figure S4: Gene expression profiles at both the genome level and the splicing factors level for B-cell samples.**

**Figure S5: Percentage of genes, containing different introns of both up-regulated and down-regulated IRI within the same gene, with first intron retained of both T-cell and B-cell samples.**

**Table S1: Summary of demographic information for 4 controls of T-cell samples.**

**Table S2: Summary of IRI distribution attributes of T-cell samples.**

**Table S3: Summary of IRI distribution attributes of B-cell samples.**

**Table S4: List of most influential genes in PC1 for both T-cell and B-cell IRI PCA.**

**Table S5: Hypergeometric probabilities of Lupus related diseases.**

**Table S6: Hypergeometric probabilities of RNA-processing functions.**

**Table S7: List of clustered genes clustering in gene expression based hierarchical clustering.**

**Table S8: List of clustered genes clustering in splicing factor expression based hierarchical clustering.**

**Table S9: List of genes in T cells that contain different introns of both up-regulated and down-regulated IRI within the same gene.**

**Table S10: List of genes in B cells that contain different introns of both up-regulated and down-regulated IRI within the same gene.**

**Figure S1. IRI distribution comparison of T-cell samples.** (A) IRI density distribution for all 18 T-cell samples. There is an upward shift in the distribution for 4 control samples (N090, N098, N048, N068) and 5 SLE patients (L140, L149, L062, L137, L072) relative to the rest SLE patients. (B-E) Illustrative Q-Q plots for 4 samples (L062, N068, L078, and L027). The Q-Q plots indicate that the IRI distributions of L062 and N068 are closer to the normal-distributions than those of L078 and L027.

**Figure S2**. **IRI distribution comparison of B-cell samples.** (A) IRI density distributions for all 20 B-cell samples. There is an upward shift in the distribution for 4 control samples relative to 16 SLE patients. (B-E) Illustrative Q-Q plots for 4 samples including 2 controls, SRR6761457 and SRR6761432, and 2 SLE patients, SRR6761419 and SRR6761423. Q-Q plots show that IRI distributions for the two SLE patients are closer to the normal distributions than those of the two controls.

**Figure S3. Mean distribution of IRI distributions of T-cell and B-cell samples.** (A) Mean density distributions for T-cell CTL IRI distributions and SLE distributions in one plot. P-value using Mann–Whitney U test is 0.079. (B) Mean density distributions for B-cell CTL IRI distributions and SLE distributions in one plot. P-value using Mann–Whitney U test is 0.0004.

**Figure S4. Gene expression profiles at both the genome level and the splicing factors level for B-cell samples.** (A) Gene expression based hierarchical clustering of all genes in the whole transcriptome of all 20 B-cell samples including 4 normal controls and 16 SLE patients. Sample clustering is shown at the top with the sample label at the bottom. (B) Same clustering as in (A) but using 81 splicing factor genes (from HUGO Gene Nomenclature Committee Database) instead of the whole genome. (C) Similar clustering patterns of sample stratifications between the whole genome gene expression (top) and the splicing factor gene expression (bottom) were indicated by the color bars with the same color for the same pattern.

**Figure S5. Percentage of genes, containing different introns of both up-regulated and down-regulated IRI within the same gene, with first intron retained of both T-cell and B-cell samples.** (A) Pie chart showing percentage of genes, containing different introns of both up-regulated and down regulated IRI within the same gene, with first intron retained for T-cell samples. This kind of genes in T-cell samples are either got first intron retained or not. (B). Same as (A), but for B-cell samples. Only 2% genes got first intron retained, while 34% genes got first intron removed.

**Table S1.** **Summary of demographic information for 4 controls of T-cell samples.** Demographic information, such as race, age, and sex, of 4 controls of T-cell samples. Information was acquired from NCBI BioSample database. For example, NCBI link, https://www.ncbi.nlm.nih.gov/biosample/4007104, for control N098.

**Table S2.** **Summary of IRI distribution attributes of T-cell samples.** The mean, standard deviation, and width (twice the standard deviation) of IRI density distribution are given in log2 scale for all 18 samples. The mean values for 4 controls (N090, N098, N048, and N068) plus 4 SLE patients (L140, L149, L062, and L137) are between -5 and -7 in log2 scale. The mean values for the rest of SLE patients are between -7 and -9 in log2 scale. Overall, the distributions are slightly wider in the latter group.

**Table S3.** **Summary of IRI distribution attributes of B-cell samples.** The mean values for 4 controls are between -3 and -5 in log2 scale while those for SLE patients are between -4 and -7 in log2 scale. Overall, the distributions for the patients are slightly wider than those for the controls.

**Table S4. List of most influential genes in PC1 for both T-cell and B-cell IRI PCA.** Specifically, 50 genes with the most negative coefficients and 100 genes with the most positive coefficients in PC1 for T or B cells were listed together with their number of occurrences as annotated to be involved in RNA-processing functions (**Table 4**) or lupus related diseases (**Table 5**).

**Table S5. Hypergeometric probabilities of Lupus related diseases.** The potential enrichment of lupus related diseases was analyzed for four subsets of genes: the top 50 genes with the most negative coefficients in PC1 for T cells (T-Negative50) or B cells (B-Negative50); and the top 100 genes with the most positive coefficients in PC1 for T (T-Positive100) or B cells (B-Positive100). The diseases that are enriched, i.e., with a p-value (the hypergeometric probability) less than 0.05 are highlighted in color.

**Table S6. Hypergeometric probabilities of RNA-processing functions.** The potential enrichment of the biological functions of RNA processing was analyzed for four subsets of genes: the top 50 genes with the most negative coefficients in PC1 for T cells (T-Negative50) or B cells (B-Negative50); and the top 100 genes with the most positive coefficients in PC1 for T (T-Positive100) or B cells (B-Positive100). The functions that are enriched, i.e., with a p-value (the hypergeometric probability) less than 0.05 are highlighted in color.

**Table S7. List of clustered genes in gene expression based hierarchical clustering.** The genes cluster returned by hierarchical clustering. Genes shown on right side of the heatmap were as location indicators to read the gene cluster table.

**Table S8. List of clustered genes in splicing factor expression based hierarchical clustering.** The spliceosome cluster used to generate hierarchical clustering heatmap. Splicing factors shown on right side of the heatmap were as location indicators to read the spliceosome cluster table.

**Table S9. List of genes in T cells that contain different introns of both up-regulated and down-regulated IRI within the same gene.** CIR_Index_Up (Down) indicates the intron index whose splicing ratio is upregulated (downregulated) when comparing SLE patients to the controls.

**Table S10. List of genes in B cells that contain different introns with both up-regulated and down-regulated IRI within the same gene.** CIR_Index_Up (Down) indicates the intron index whose splicing ratio is upregulated (downregulated) when comparing SLE patients to the controls.
