# Supplementary figures and images for: Abnormalities in intron retention characterize patients with systemic lupus erythematosus
Source: Sci Rep. 2023 Mar 29;13:5141. doi: 10.1038/s41598-023-31890-4 (PMC10060252; doi:10.1038/s41598-023-31890-4)

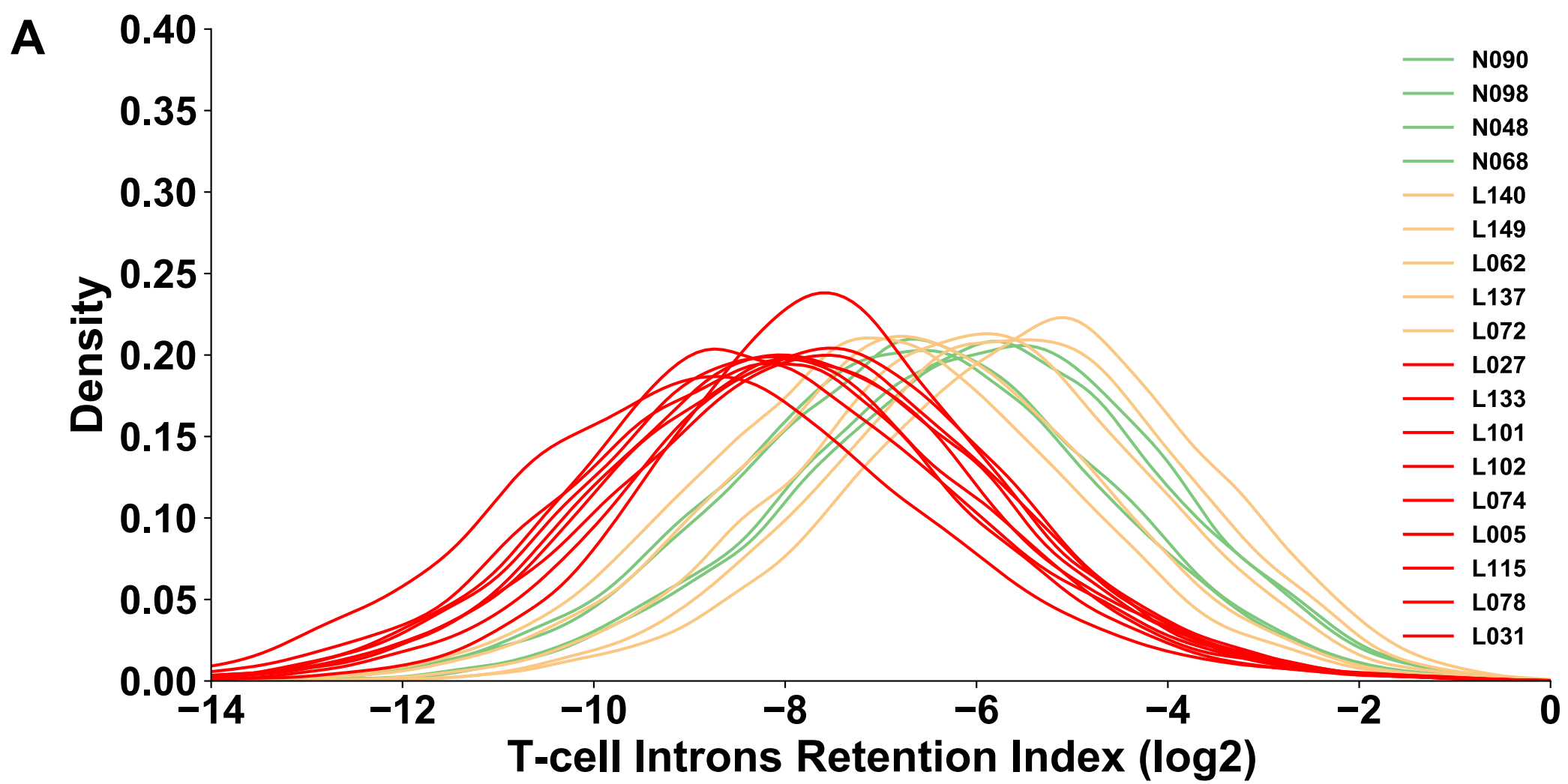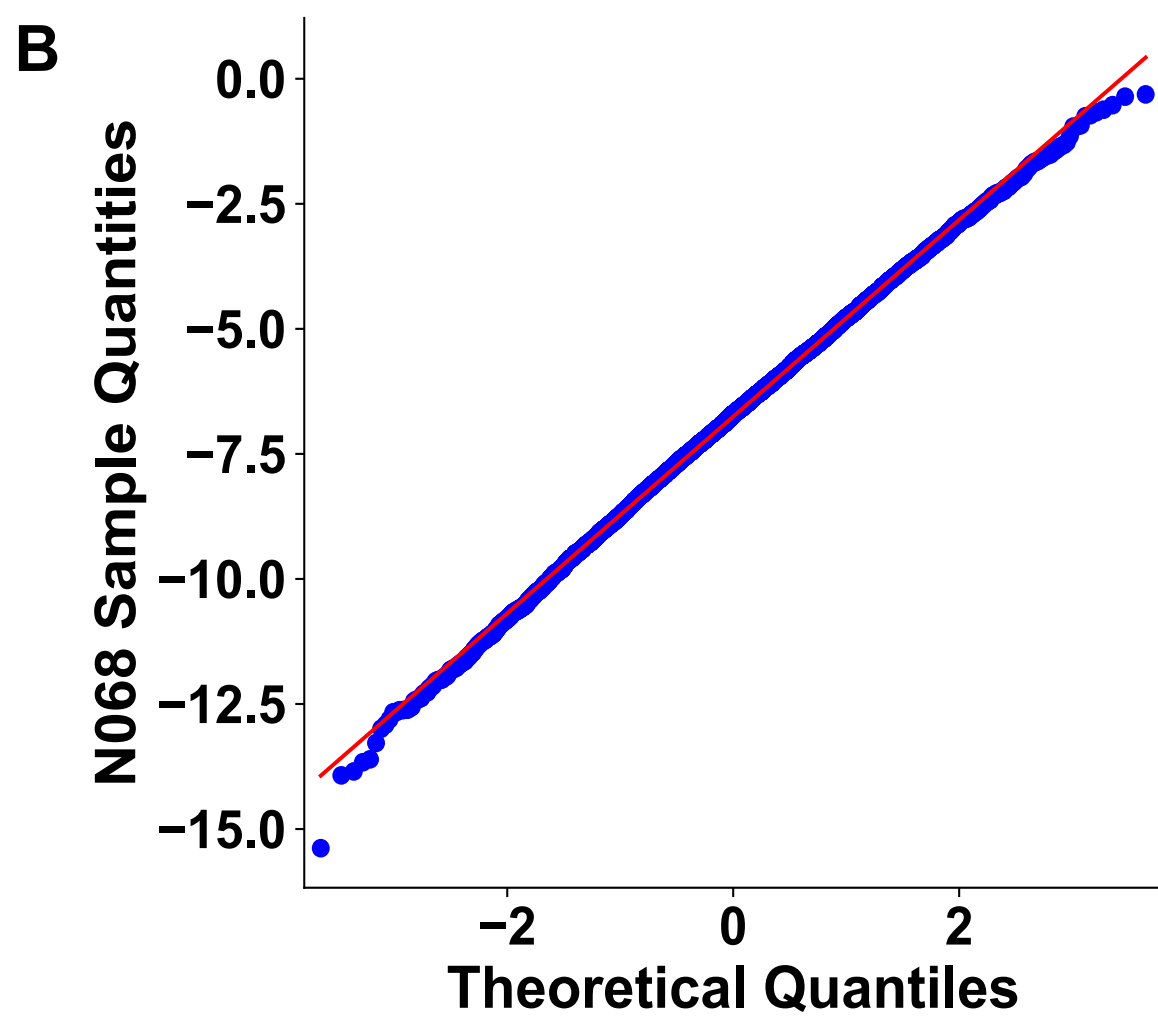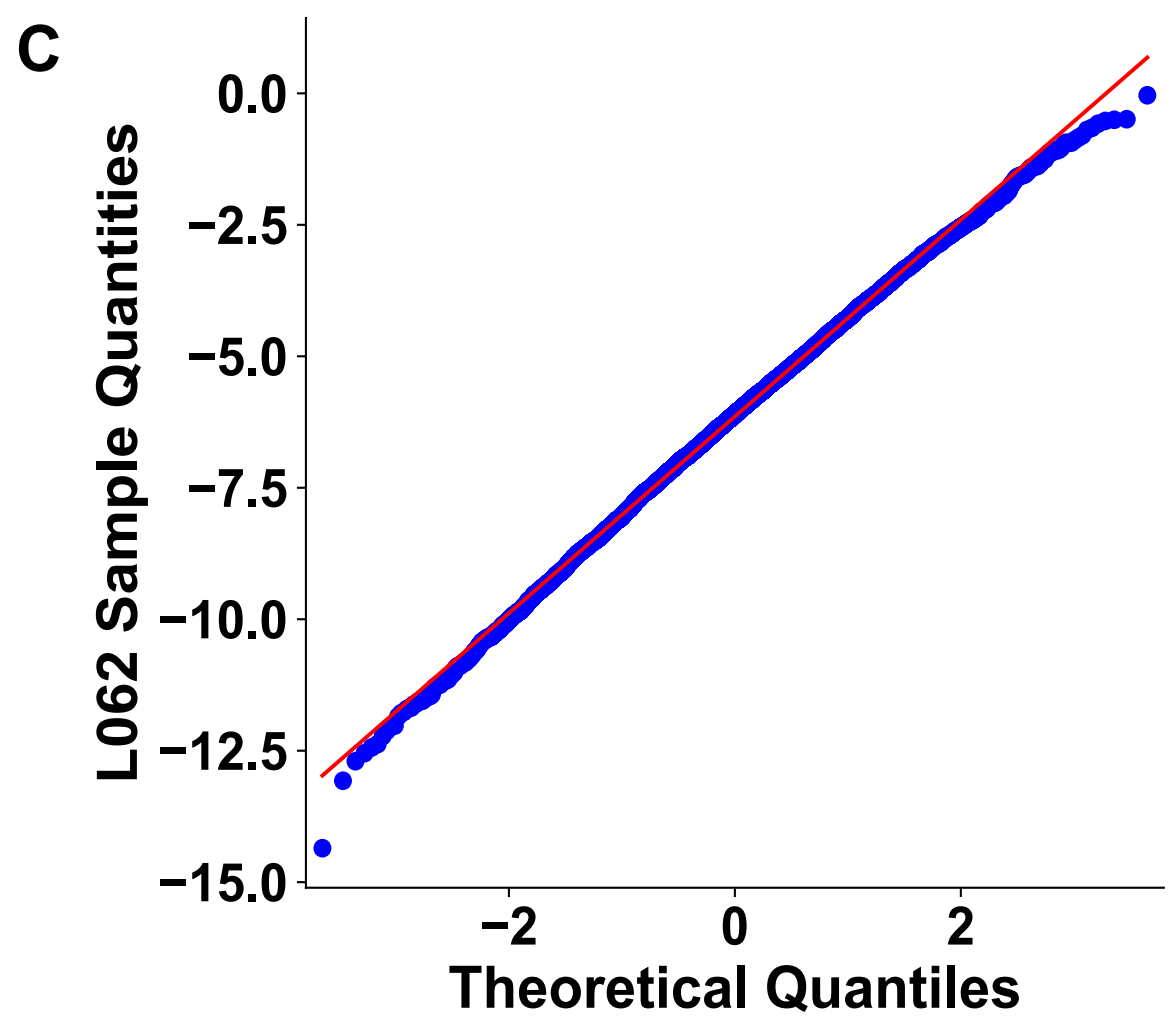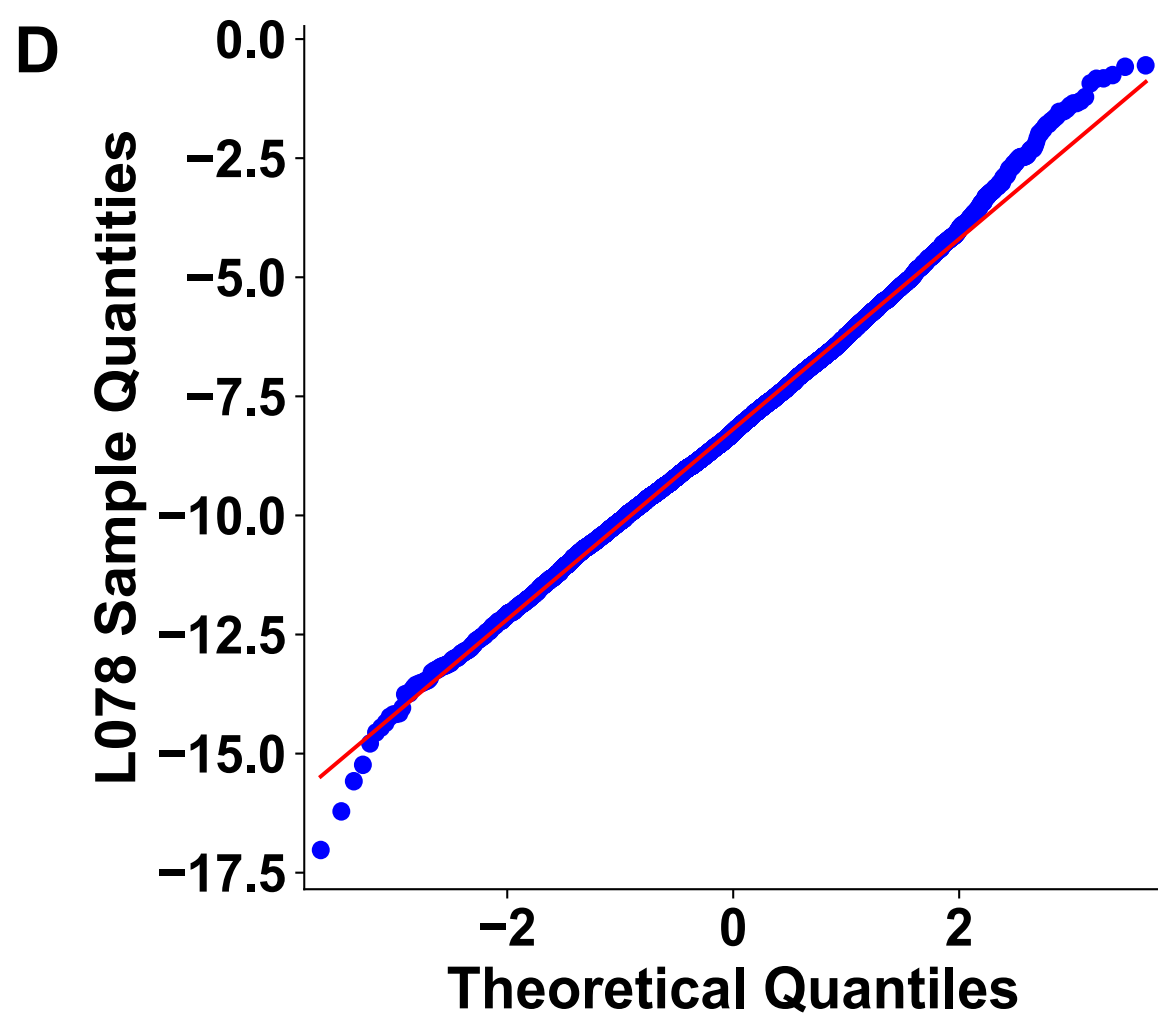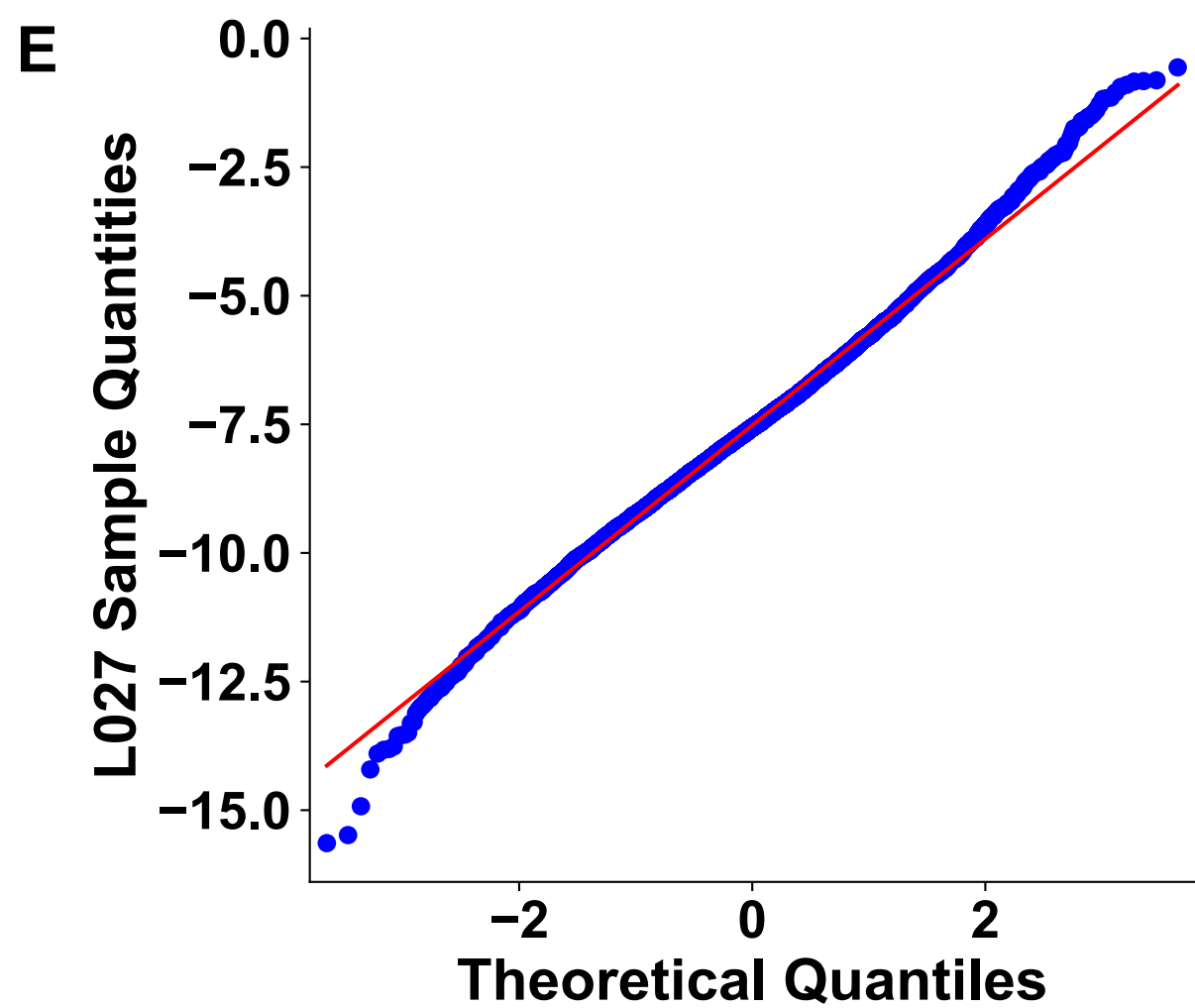

Supplement: Supplementary file 2 — Supplementary Figure S1. [file 41598_2023_31890_MOESM2_ESM.pdf]

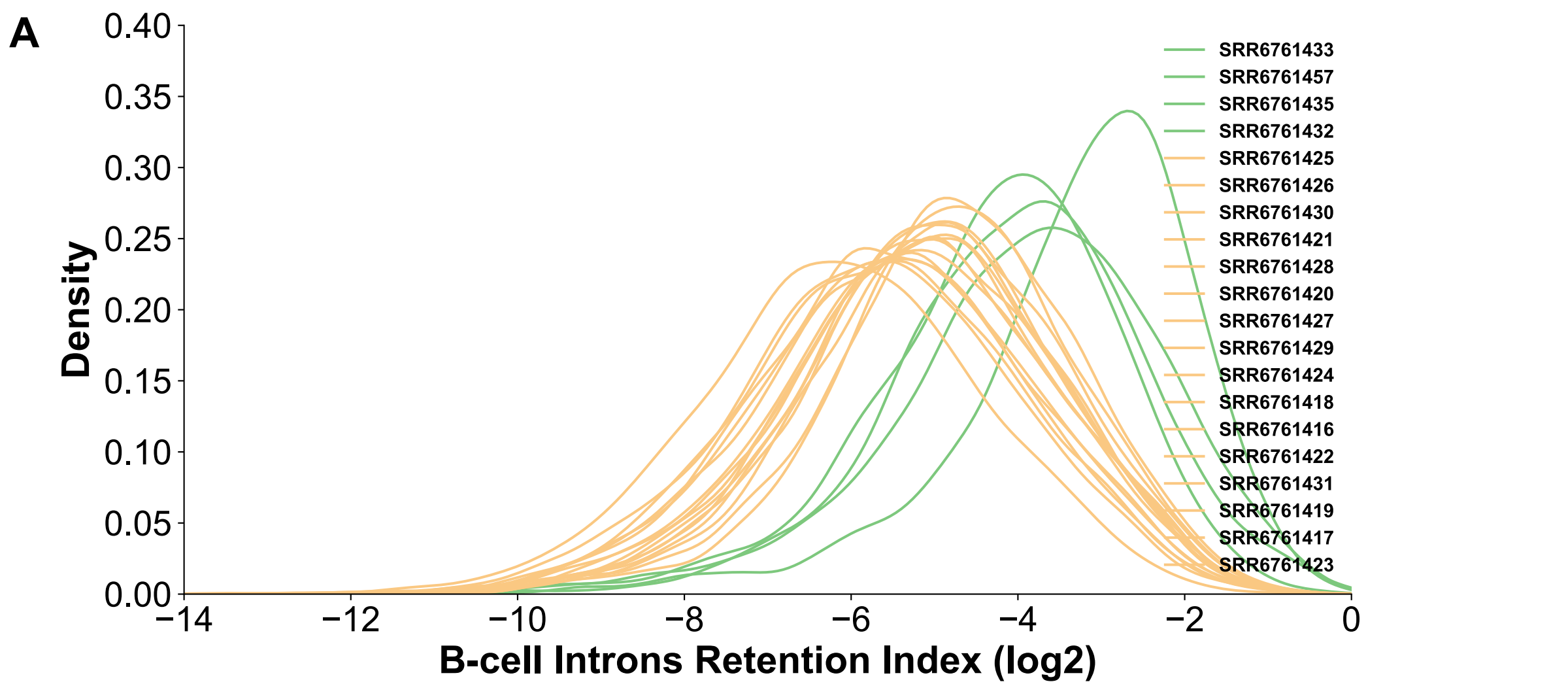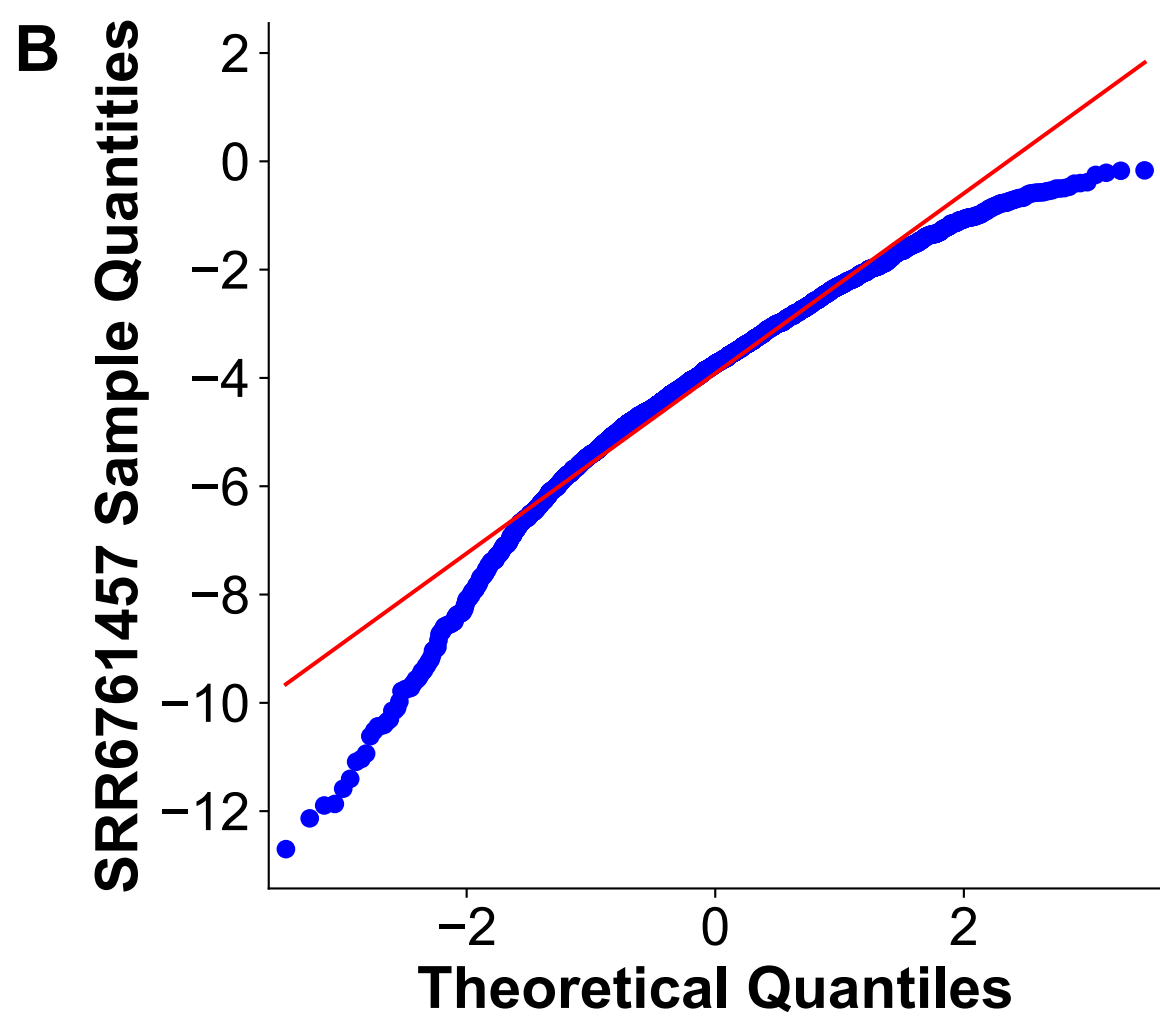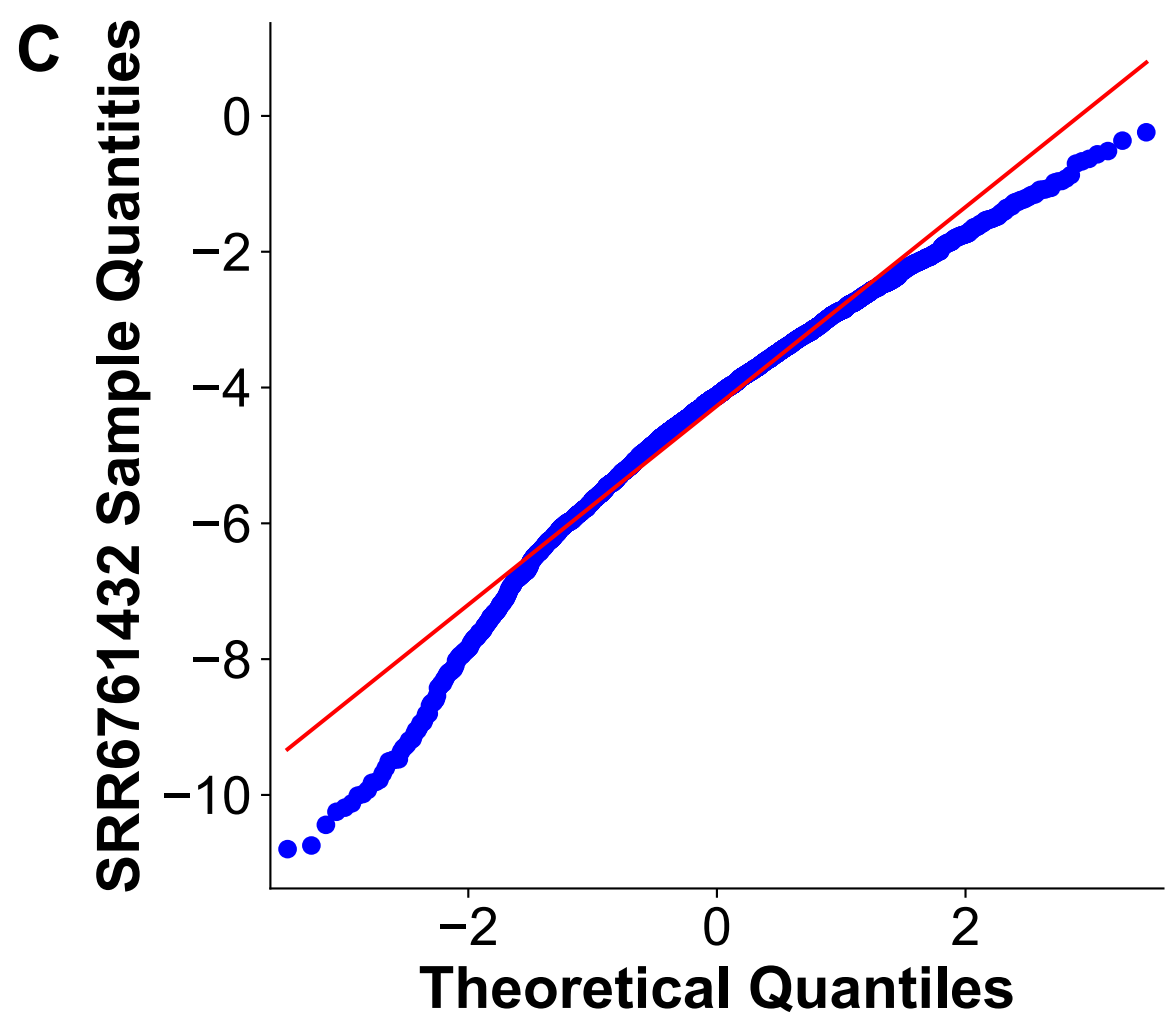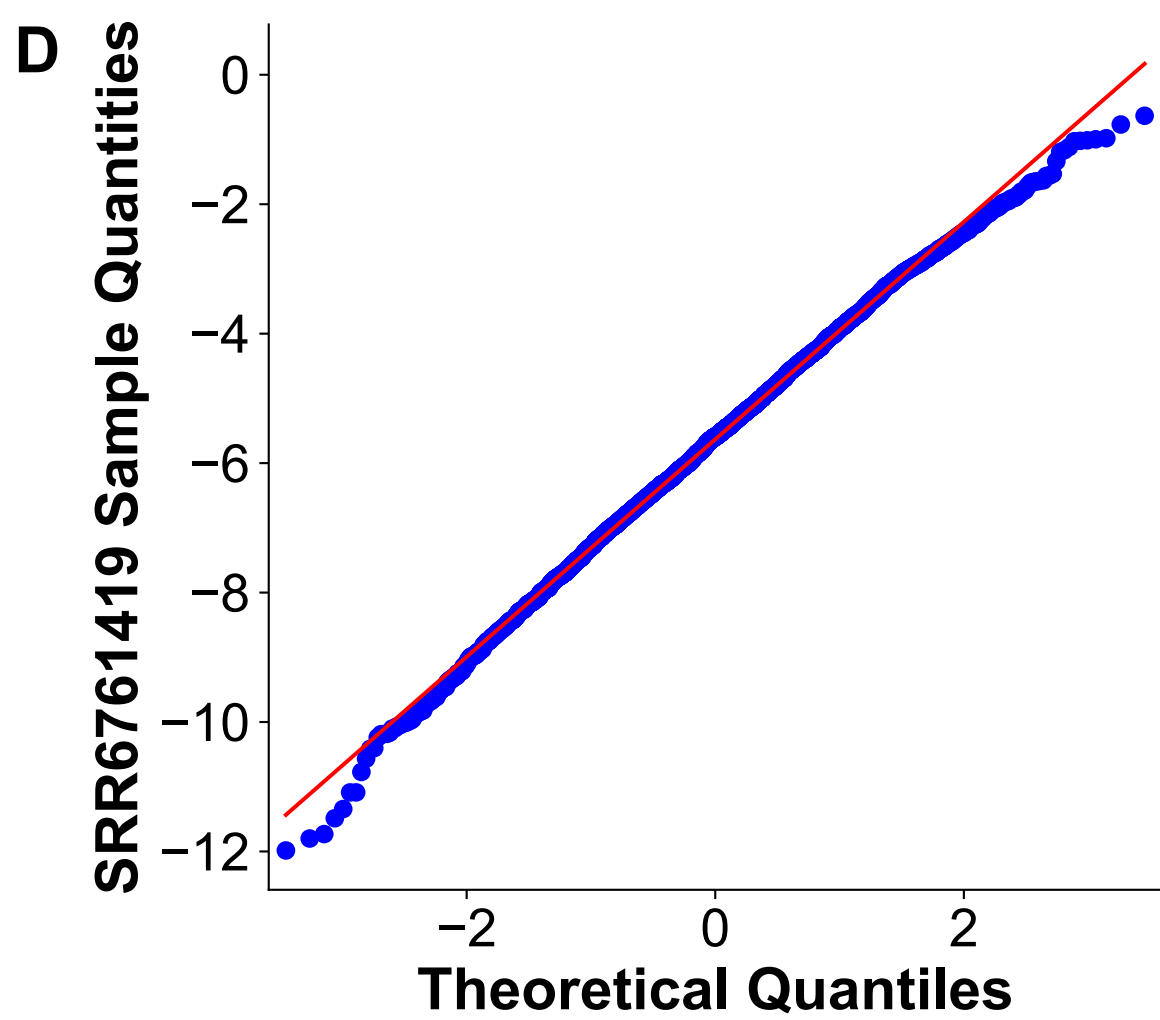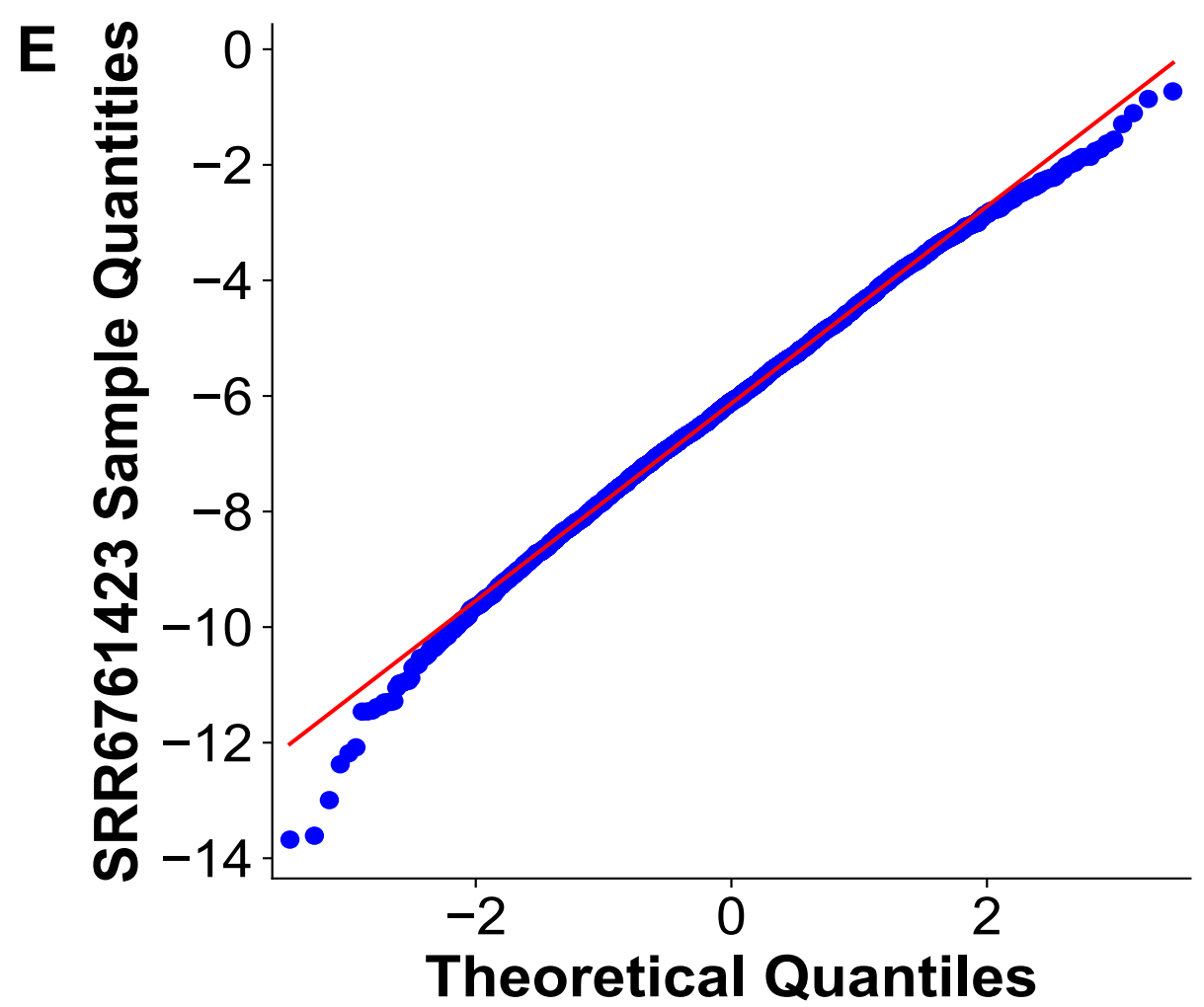

Supplement: Supplementary file 3 — Supplementary Figure S2. [file 41598_2023_31890_MOESM3_ESM.pdf]

**A****T cell**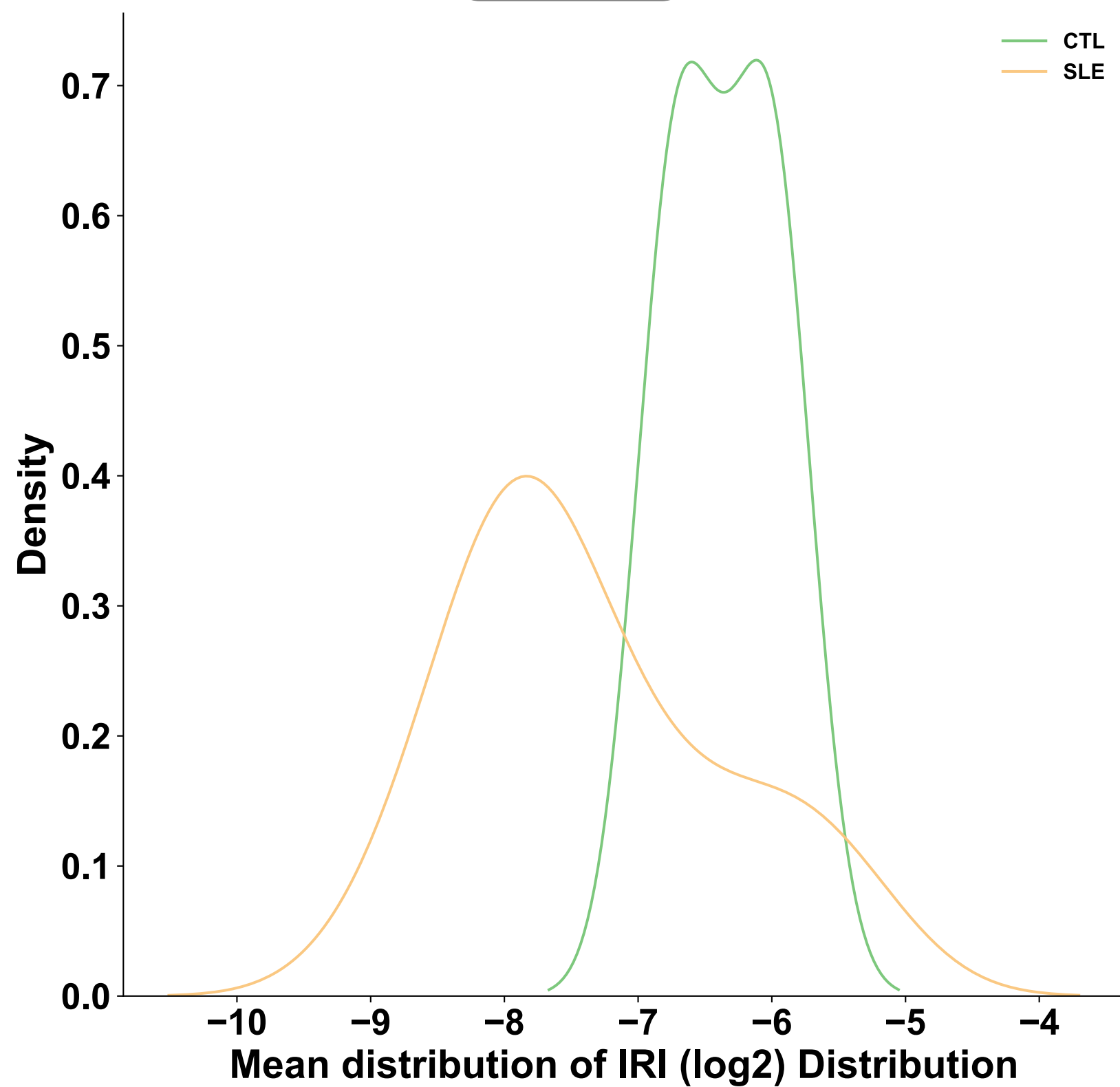**B****B cell**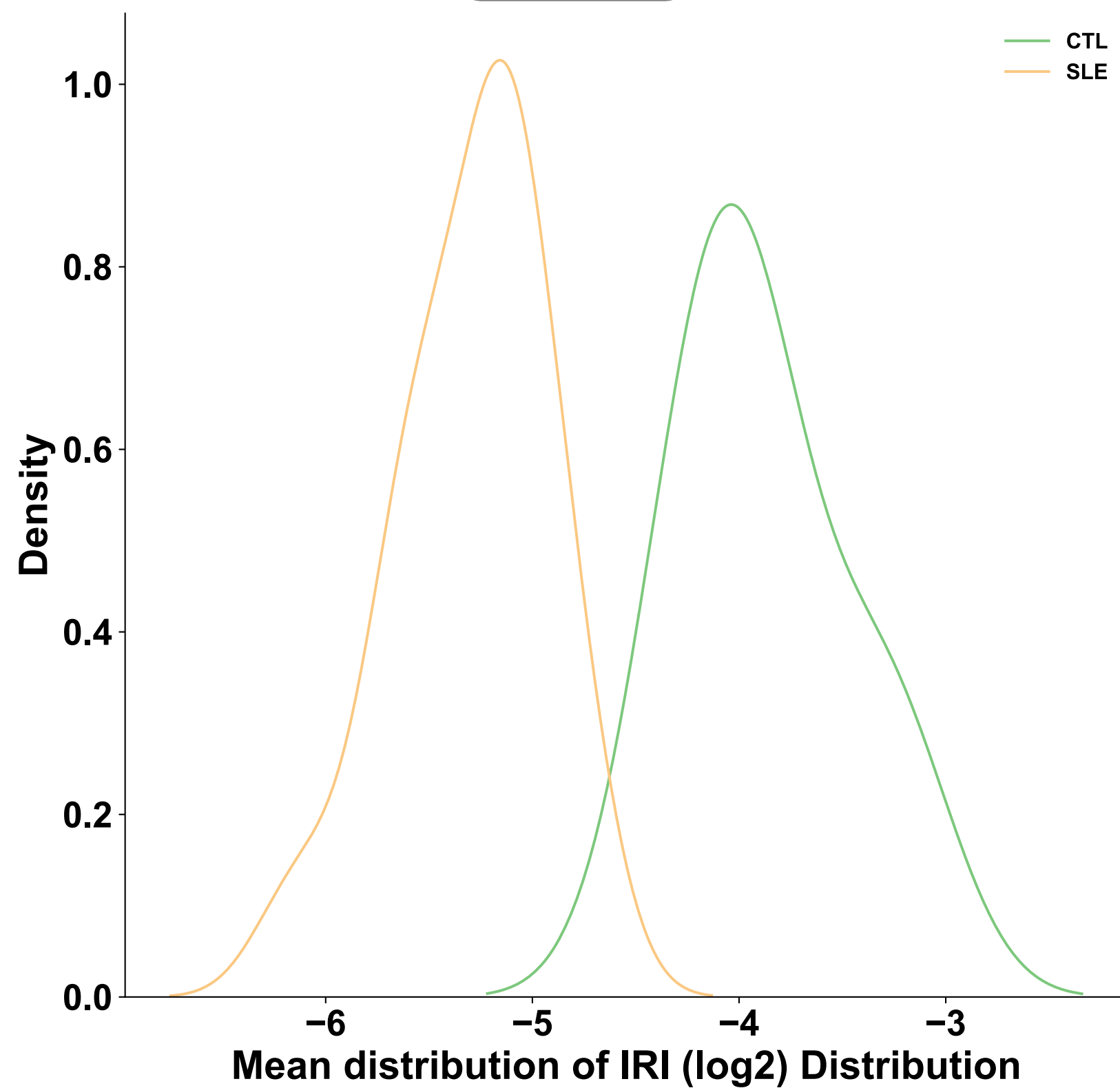

Supplement: Supplementary file 4 — Supplementary Figure S3. [file 41598_2023_31890_MOESM4_ESM.pdf]

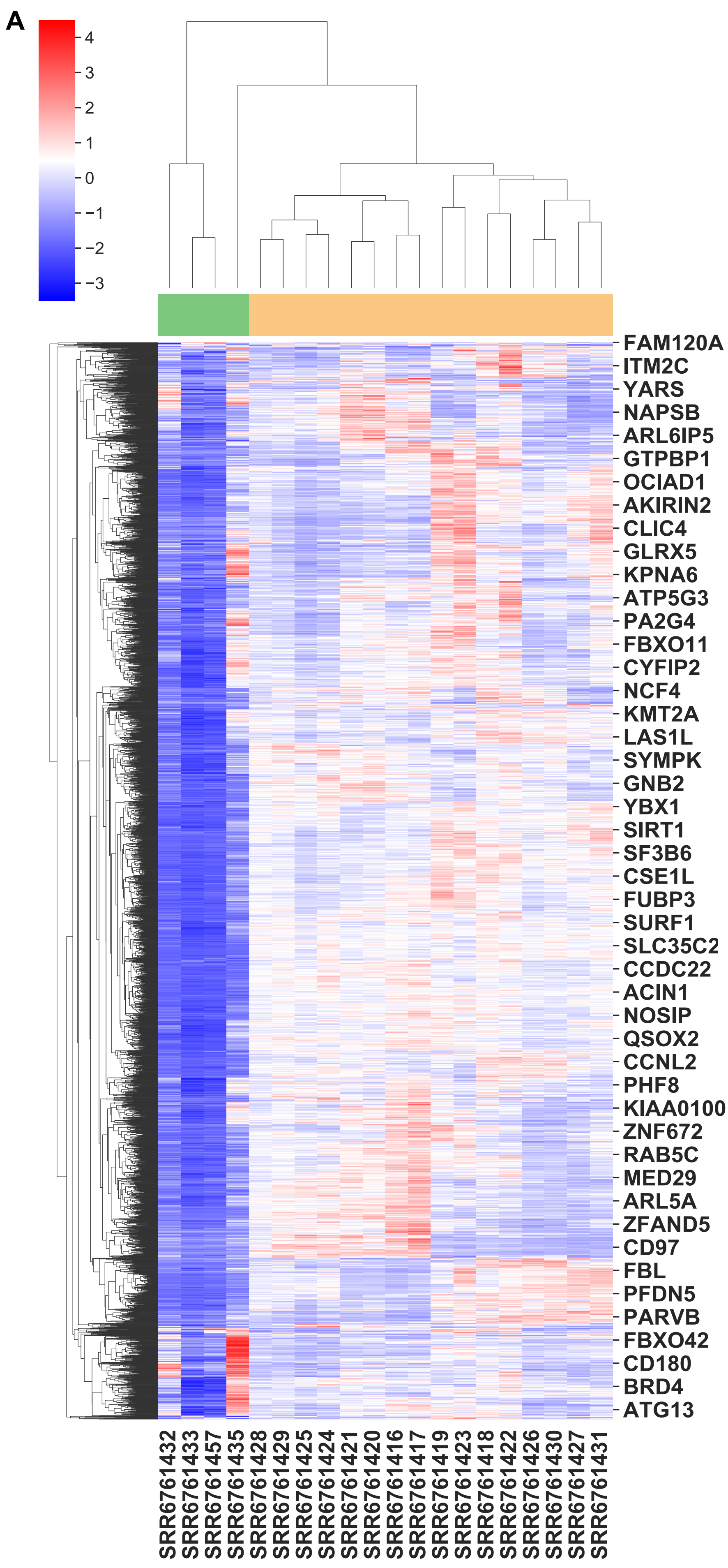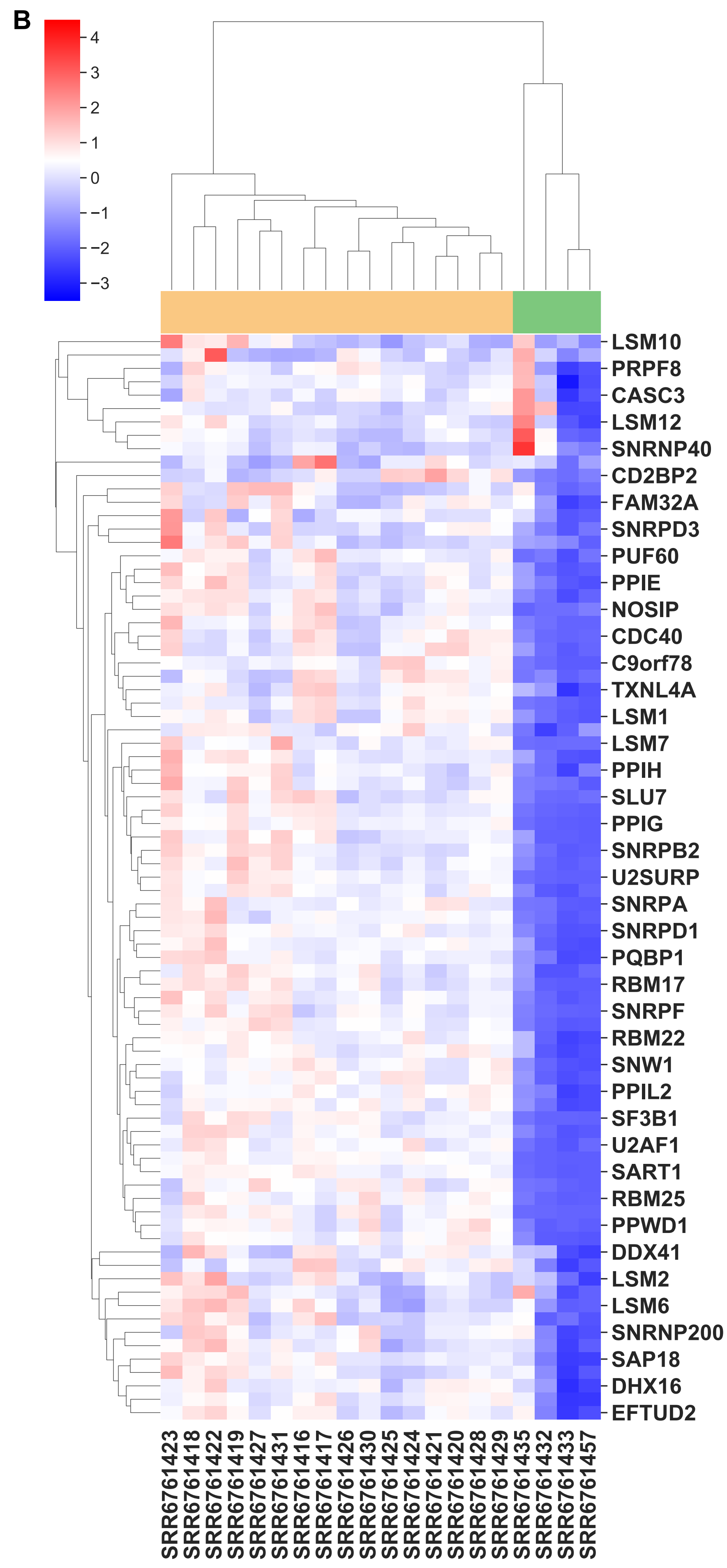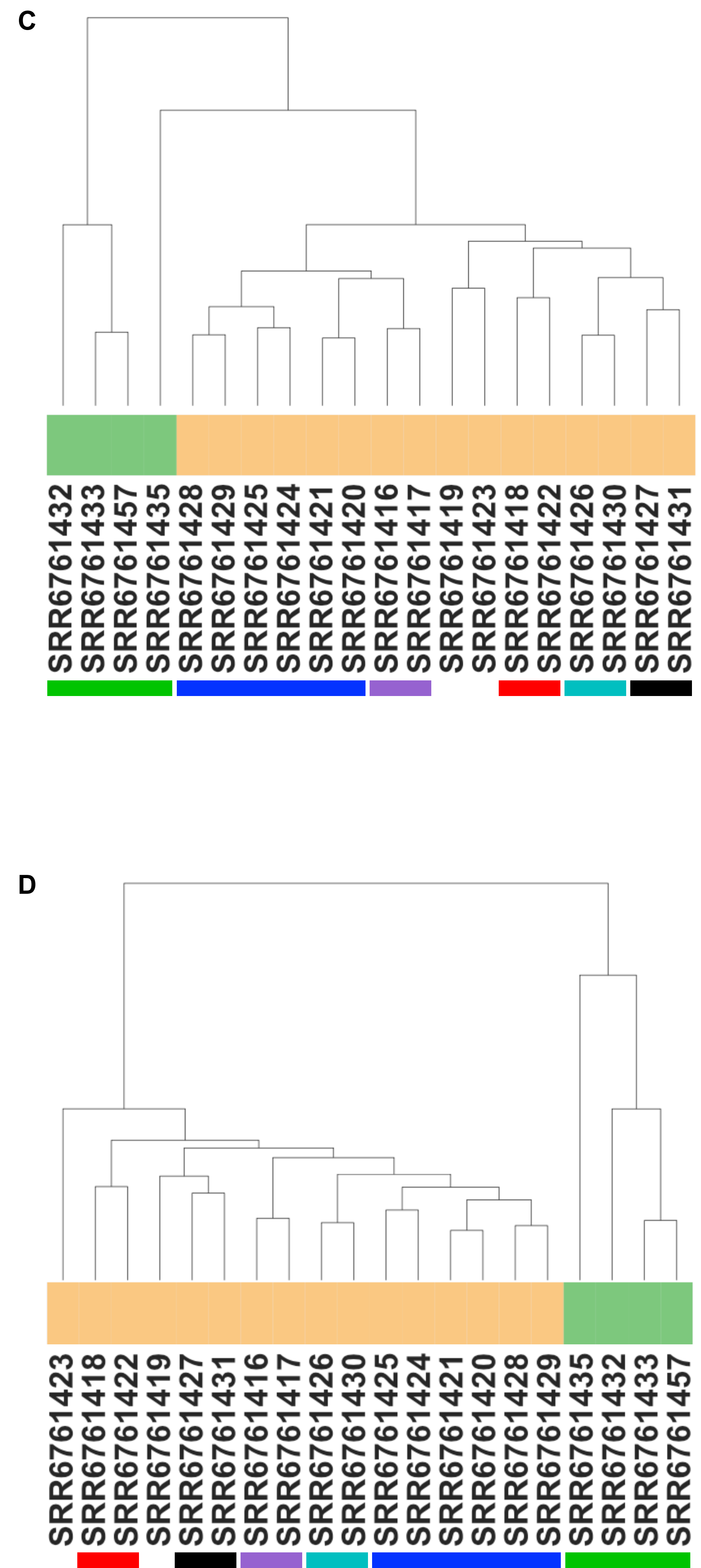

Supplement: Supplementary file 5 — Supplementary Figure S4. [file 41598_2023_31890_MOESM5_ESM.pdf]

**A**

**T cell**

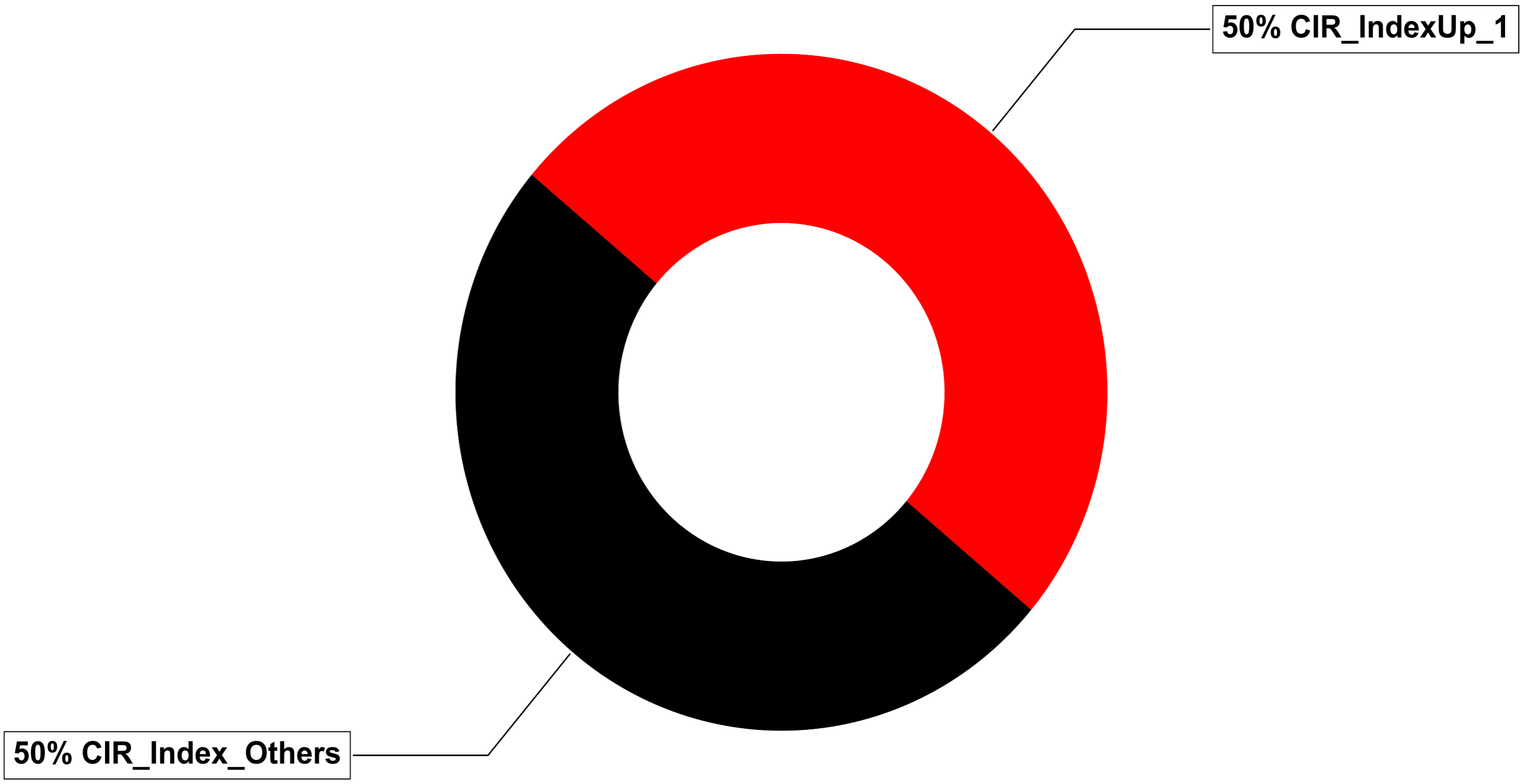

**B**

**B cell**

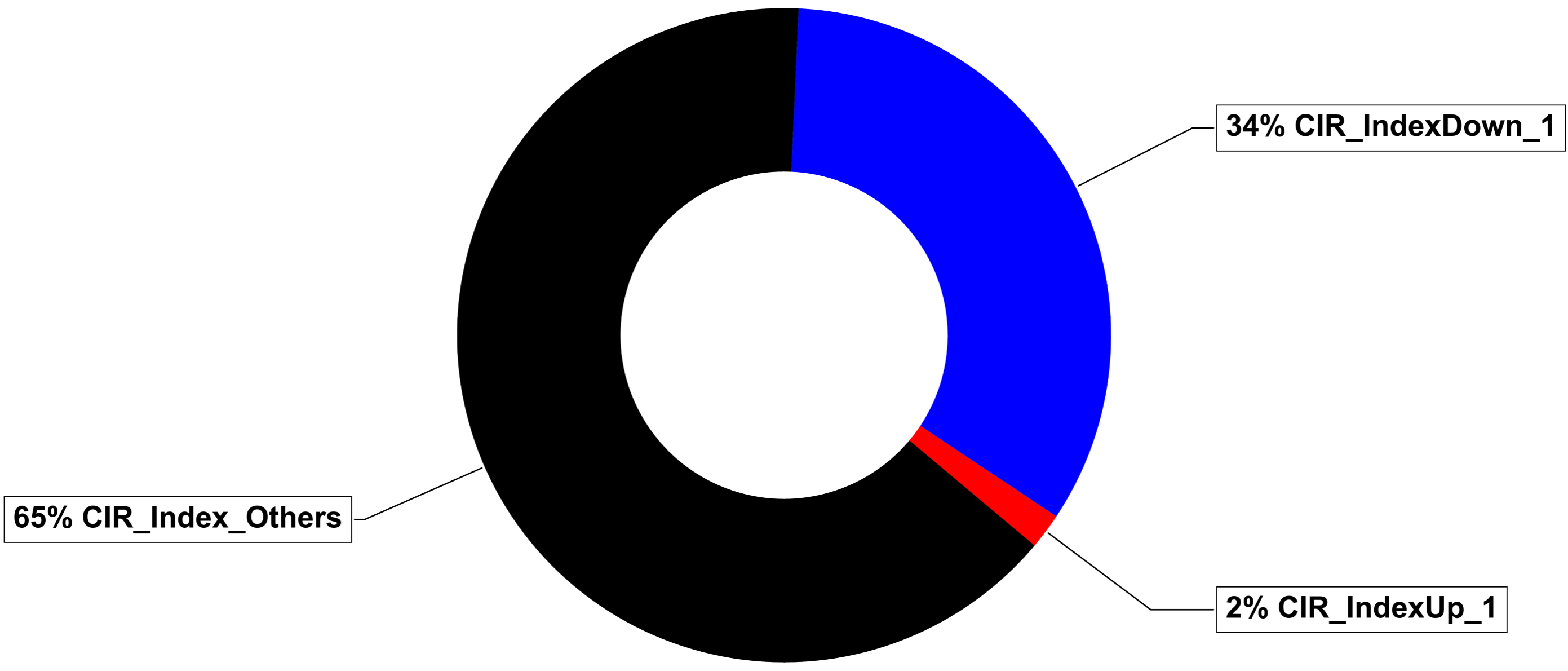

Supplement: Supplementary file 6 — Supplementary Figure S5. [file 41598_2023_31890_MOESM6_ESM.pdf]
